# Supplementary material for: Vav2 catalysis-dependent pathways contribute to skeletal muscle growth and metabolic homeostasis
Source: Nat Commun. 2020 Nov 16;11:5808. doi: 10.1038/s41467-020-19489-z (PMC7669868; doi:10.1038/s41467-020-19489-z)
Supplement: Supplementary file 1 — Supplementary Information [file 41467_2020_19489_MOESM1_ESM.pdf]

Supplementary Information for

Vav2 CATALYSIS-DEPENDENT PATHWAYS CONTRIBUTE TO SKELETAL MUSCLE GROWH AND METABOLIC HOMEOSTASIS

by

Rodríguez-Fdez *et al*.

This PDF file includes:

(1) Supplementary Figures 1 to 18 and legends (pages 2 to 29)

(2) Supplementary Tables 1 and 2 (pages 30 to 31)

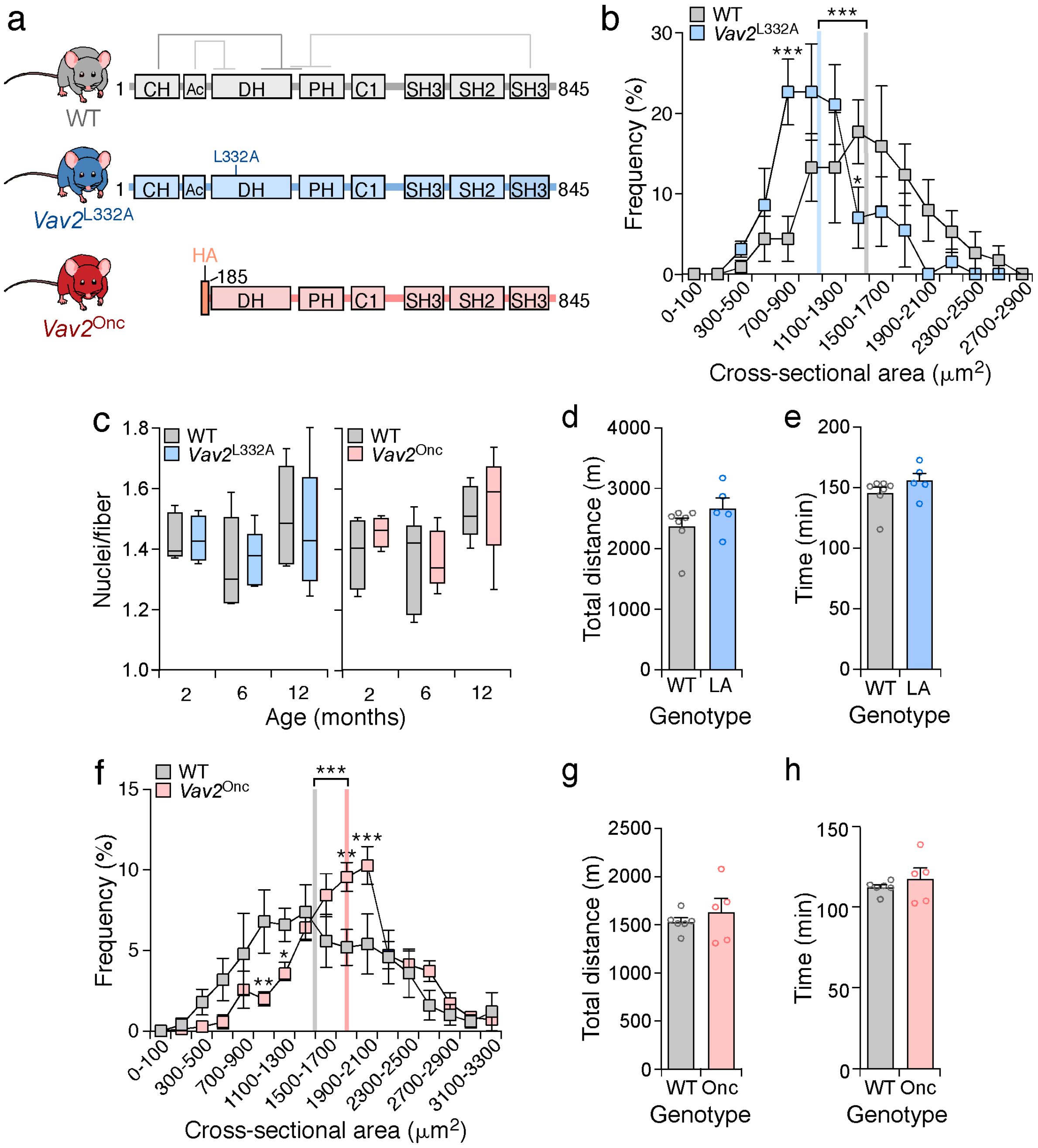

SUPPLEMENTARY FIGURE 1. The Vav2 catalytic output affects the fiber size but not the function of skeletal muscle

(a) Scheme of the domain structure of Vav2. We also show the known intramolecular regulation of Vav family proteins (top) that regulate their catalytic activities and the mutant alleles used in our mouse studies. CH, calponin-homology; Ac, acidic domain; DH, Dbl-homology; PH, pleckstrin-homology; C1, zinc finger of the C1 subtype; SH, Src-homology.

(b) Distribution of the cross-sectional area of the fibers in the gastrocnemius muscle of 12-month-old animals of the indicated genotypes (inset). Values are presented as mean ± SEM. Light gray and blue lines indicate the mean cross-sectional fiber area in WT and *Vav2*$^{L332A/L332A}$ mice, respectively. *, $P = 0.02$; ***, $P = 0.0004$ using

two-tailed Student's t tests (in the case of the mean) and two-way ANOVA followed by Fisher's LSD tests. n = 5 (WT) and 6 (*Vav2*$^{L332A/L332A}$) mice.

(c) Number of nuclei per myofiber from mice of the indicated ages (bottom) and genotypes (inset). Boxes, lines inside boxes, and bars represent the 25th to 75th percentiles, the median, and the minimum and maximum values, respectively. n = 4 (2-month-old *Vav2*$^{Onc/Onc}$ mice and their WT controls, 2-month-old *Vav2*$^{L332A/L332A}$ mice), 5 (2-month-old WT controls for the *Vav2*$^{L332A/L332A}$ mice, 6- and 12-month-old *Vav2*$^{L332A/}$L332A mice and their controls, 6-month-old *Vav2*$^{Onc/Onc}$ mice and 12-month-old controls for the *Vav2*$^{Onc/Onc}$ mice), 6 (6-month-old WT controls for the *Vav2*$^{Onc/Onc}$ mice) and 7 (12-month-old *Vav2*$^{Onc/Onc}$ mice).

(d,e) Total distance (d) and time (e) run by 3-month-old mice of the indicated genotype in a treadmill challenge. Data are shown as mean ± SEM. n = 7 WT and 5 *Vav2*$^{L332A/L332A}$ animals.

(f) Distribution of the cross-sectional area of the fibers in the gastrocnemius muscle of 12-month-old animals of the indicated genotypes (inset). Values are presented as mean ± SEM. Light gray and red lines indicate the mean cross-sectional fiber area in WT and *Vav2*$^{Onc/Onc}$ mice, respectively. *, *P* = 0.0365; **, *P* = 0.0010 (900-1100 range) and *P* = 0.0027 (1700-1900 range); ***, *P* = 0.0008 using two-tailed Student's t tests (in the case of the mean) and two-way ANOVA followed by Fisher's LSD tests. n = 5 (WT) and 7 (*Vav2*$^{Onc/Onc}$) mice.

(g,h) Performance of 3-month-old mice of the indicated genotype in a treadmill running. Total distance (g) and time (h) run are expressed as mean ± SEM. n = 6 (WT) and 5 (*Vav2*$^{Onc/Onc}$) animals.

Source data for this figure are provided as a Source Data file.

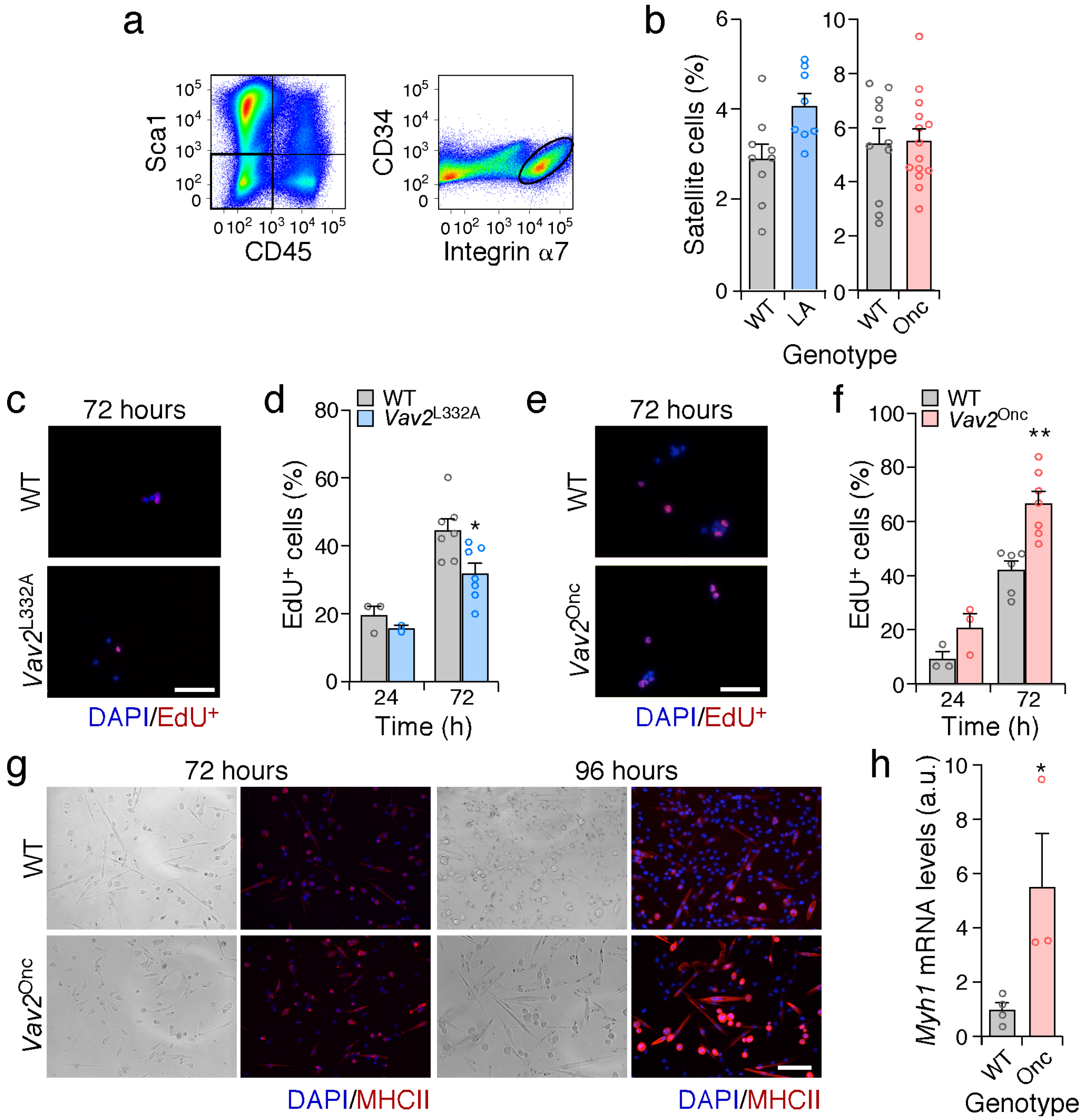

SUPPLEMENTARY FIGURE 2. Effect of Vav2 catalytic output in satellite cells

(a) Representative isolation of satellite cells from mouse hind-limb muscles using fluorescence activated cell sorting. After negative selection of the Sca1$^+$;CD45$^+$ cells expressing (left panel), only the CD34$^+$ and integrin α7$^+$ double positive cells were purified and cultured.

(b) Percentage of satellite cells in the hind-limb muscles from 3-month-old animals of the indicated genotypes (bottom). LA: *Vav2*$^{L332A/L332A}$; Onc: *Vav2*$^{Onc/Onc}$. Values are presented as mean ± SEM. n = 9 (WT control for *Vav2*$^{L332A/L332A}$), 8 (*Vav2*$^{L332A/L332A}$), 11 (WT control for *Vav2*$^{Onc/Onc}$) and 14 (*Vav2*$^{Onc/Onc}$) mice.

(c-f) Representative image (c,e) and quantification (d,f) of the percentage of EdU positive satellite cells of the indicated genotypes and culture times. Data represent the mean ± SEM. *, P = 0.0138; **, P =0.0013 using two-tailed Student's t tests. n = 3 (24 hours time-point), 7 (WT controls for *Vav2*$^{L332A/L332A}$, *Vav2*$^{L332A/L332A}$, and *Vav2*$^{Onc/Onc}$ mice; 72 hours time-point), and 6 (WT controls for *Vav2*$^{Onc/Onc}$ mice, 72 hours time-point). Scale bars in c and e, 50 µm.

(g) Representative light and immunofluorescence images of satellite cells stained with an antibody to myosin heavy chain II (MHCII, red color) and 4',6-Diamidine-2'-phenylindole dihydrochloride (DAPI, blue color). n = 7 (WT) and 10 (*Vav2*$^{Onc/Onc}$) independent cell cultures. Scale bar, 50 µm.

(h) Relative expression of the *Myh1* mRNA in satellite cells that were maintained under differentiation culture conditions for 96 hours. The expression obtained in WT cells was given an arbitrary value of 1. Data are shown as mean ± SEM. *, *P* = 0.0446 using two-tailed Student's *t* tests. n = 4 (WT) and 3 (*Vav2*$^{Onc/Onc}$) independent satellite cell cultures. a.u., arbitrary units.

Source data for this figure are provided as a Source Data file.

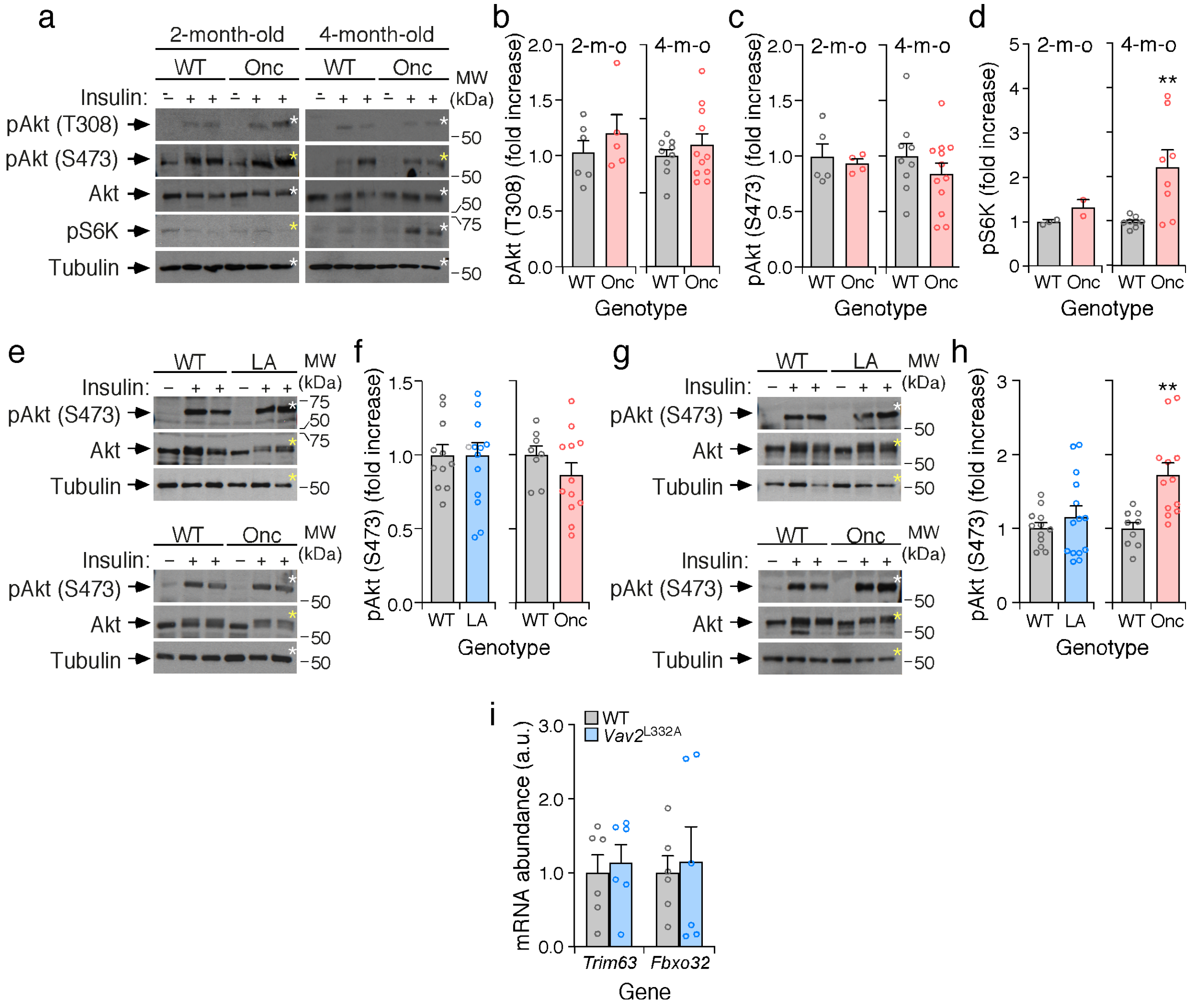

SUPPLEMENTARY FIGURE 3. The Vav2 catalytic output does not affect liver or WAT insulin signaling

(a-d) Representative immunoblots (a) and quantification of the phosphorylation levels of indicated proteins and phosphosites (b-d, left) in gastrocnemius muscles from WT and Vav2$^{Onc/Onc}$ (Onc) mice of the indicated ages (top) that were either untreated (–) or treated (+) with insulin for 5 min. In b-d, data are presented as mean ± SEM. **, $P = 0.0071$ using two-tailed Student's t tests. n = 6 (2-month-old WT mice, b and c), 5 (2-month-old Vav2$^{Onc/Onc}$ animals, b and c), 9 (4-month-old WT mice, b and c), 11 (4-month-old *Vav2*$^{Onc/Onc}$ animals, b and c), 3 (2-month-old mice, d) or 8 (4-month-old mice, d). In each case, samples were run in parallel on two gels (the same filter is indicated with asterisks of the same colour).

(e,f) Representative immunoblots (e) and quantification (f) of the phosphorylation levels of indicated proteins and phosphosites in liver from 4-month-old WT, *Vav2*$^{L332A/L332A}$ (LA), and *Vav2*$^{Onc/Onc}$ (Onc) mice that were infused with either placebo (–) or insulin (+) for 2 min. In e, tubulin was used as loading control. Aliquots from the same lysates were analyzed in separate blots (each identified with same color asterisks). In f, data are presented as mean ± SEM. n = 11 (WT controls for *Vav2*$^{L332A/L332A}$ animals), 12 (*Vav2*$^{Onc/Onc}$ mice), 14 (*Vav2*$^{L332A/L332A}$ animals) and 8 (WT controls for *Vav2*$^{Onc/Onc}$ mice). In each case, samples were run in parallel on two gels (the same filter is indicated with asterisks of the same colour).

(g,h) Representative immunoblots (g) and quantification (h) of phospho-Akt levels in the gonadal adipose tissue of animals of the indicated genotypes that were infused with either placebo (–) or insulin (+) for 2 min. In g, tubulin was used as loading control. Aliquots from the same lysates were analyzed in separate blots (each identified with same color asterisks). In h, data are presented as mean ± SEM. **, $P = 0.0021$ using two-tailed Student's *t* tests. n = 12 (WT controls for *Vav2*$^{L332A/L332A}$ animals and *Vav2*$^{Onc/Onc}$ mice), 14 (*Vav2*$^{L332A/L332A}$

animals), and 9 (WT for *Vav2*$^{Onc/Onc}$ mice). In each case, samples were run in parallel on two gels (the same filter is indicated with asterisks of the same colour).

(i) Relative abundance of the indicated transcripts in the gastrocnemius from 3-month-old mice of indicated genotypes (n = 6 animals per group).

Source data for this figure are provided as a Source Data file.

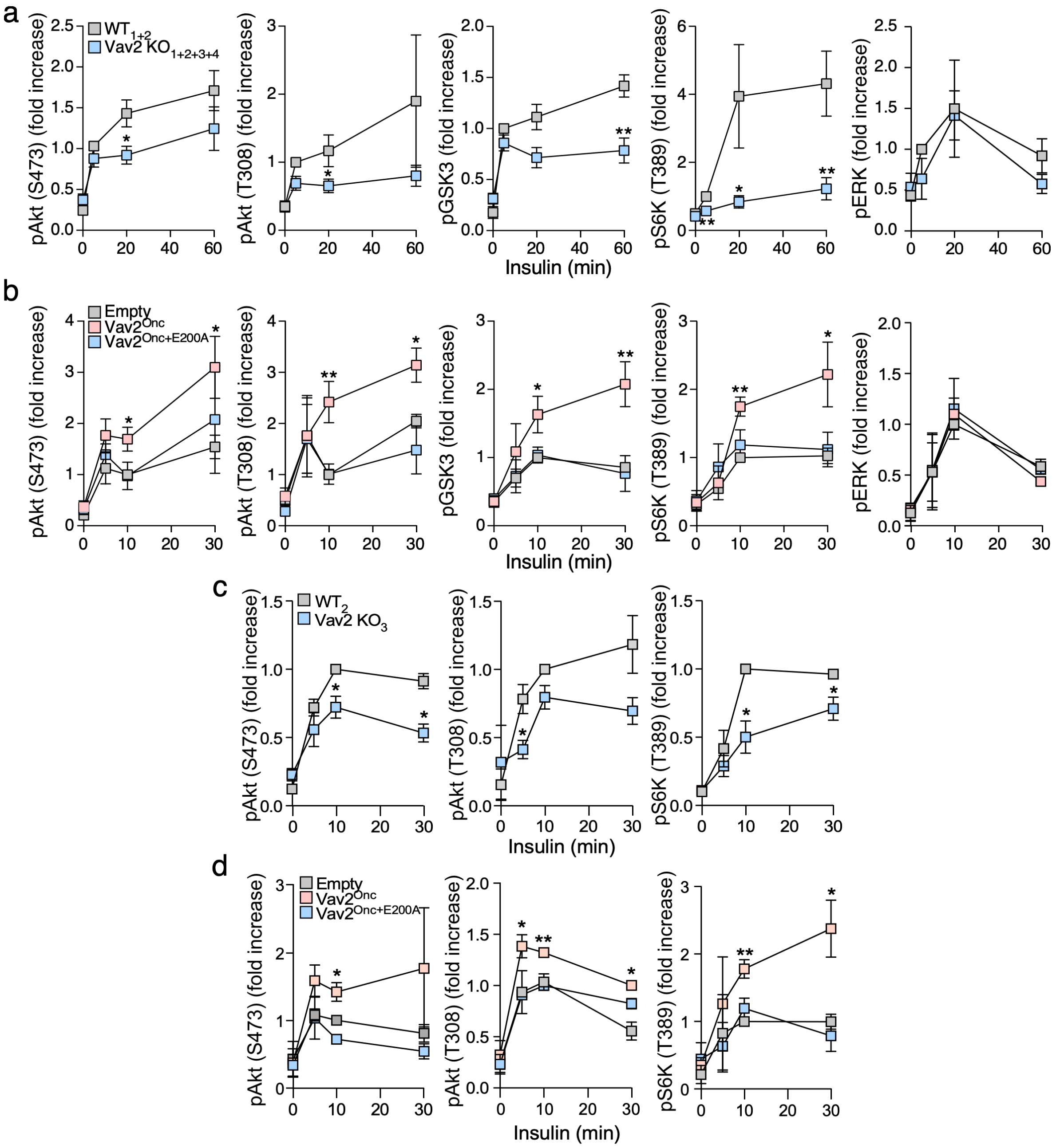

SUPPLEMENTARY FIGURE 4. The Vav2 catalytic output affects insulin response in C2C12 cells

(a,b) Quantification of the phosphorylation levels of indicated proteins and phosphosites in *Vav2* knockout (by gene-editing, KO) (a), Vav2^{Onc}- (b), and Vav2$^{\text{Onc+E200A}}$-expressing (b) C2C12 cells upon stimulation with 75 nM insulin for the indicated periods of time (bottom). Data are shown as mean ± SEM. *, $P = 0.0213$ (pAKT (S473), a), $P = 0.0367$ (pAKT (T308), a), $P = 0.0199$ (pS6K, a), $P = 0.0105$ (10 min, pAKT (S473), b), $P = 0.0430$ (30 min, pAKT (S473), b), $P = 0.0162$ (30 min, pAKT (T308), b), $P = 0.0399$ (10 min, pGSK3, b), and $P = 0.0400$ (30 min, pS6K, b); **, $P = 0.0045$ (pGSK3, a), $P = 0.0019$ (5 min, pS6K, a), $P = 0.0030$ (60 min, pS6K, a), $P = 0.0030$ (60 min, pS6K, a), $P = 0.0043$ (pAKT (T308), b), $P = 0.0068$ (pGSK3, b), and $P = 0.0004$ (pS6K, b) using two-tailed Student's *t* tests. n = at least 3 (a) and 4 (b) independent experiments.

(c,d) Quantification of the phosphorylation levels of indicated proteins and phosphosites in *Vav2* KO (c), Vav2^{Onc}- (d), and Vav2$^{\text{Onc+E200A}}$-expressing (d) in differentiated C2C12 cells upon stimulation with 75 nM

insulin for the indicated periods of time (bottom). Data are shown as mean ± SEM. *, $P = 0.0268$ (10 min, left panel, c), $P = 0.01156$ (30 min, left panel, c), $P = 0.0422$ (5 min, middle panel, c), $P = 0.0136$ (10 min, right panel, c), $P = 0.0490$ (30 min, right panel, c), $P = 0.0039$ (10 min, left panel, d), $P = 0.0149$ (5 min, middle panel, d), and $P = 0.0350$ (30 min, right panel, d); **, $P = 0.0042$ (10 min, middle panel, d) and $P = 0.0046$ (10 min, right panel, d) using two-tailed Student's *t* tests. n = 3 independent experiments.

Source data for this figure are provided as a Source Data file.

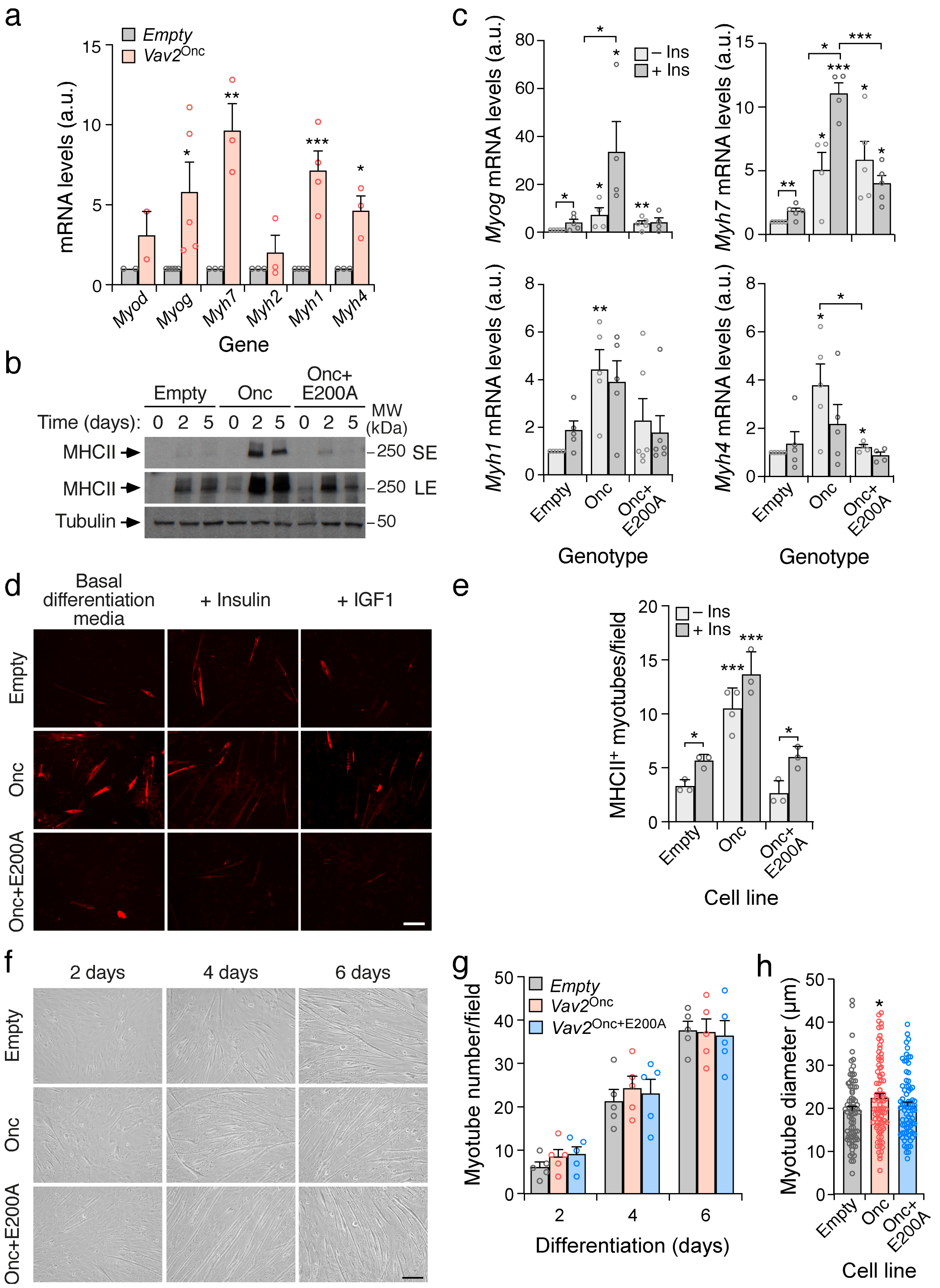

SUPPLEMENTARY FIGURE 5. Effect of Vav2^{Onc} in the differentiation of C2C12 cells

(a) Abundance of indicated differentiation-related mRNAs in the indicated cell lines upon differentiation for 3 days. Data are shown as mean ± SEM. *, $P = 0.0329$ (*Myog*) and $P = 0.0167$ (*Myh4*); **, $P = 0.0069$; ***, $P = 0.0004$ using two-tailed Student's *t* tests. n = 5 (*Myog*), 4 (*Myh1*) and 3 (other genes) independent experiments.

(b) Representative immunoblots showing the abundance of myosin heavy chain (MHCII) in the control (empty), HA-Vav2^{Onc}- (Onc), and Vav2$^{Onc+E200A}$ (Onc+E200A)-expressing C2C12 cells that were differentiated for the indicated periods of time (top). Tubulin was included as a loading control (n = 3 independent experiments). SE, short exposure of the blot. LE, long exposure of the same blot.

(c) Abundance of indicated transcripts in control (empty), HA-Vav2^{Onc}- (Onc), and Vav2$^{Onc+E200A}$ (Onc+E200A)-expressing C2C12 cells upon differentiation for 3 days either in absence or presence of insulin (Ins). Data are shown as mean ± SEM. Statistical values were calculated using two-tailed Student's *t* tests and given relative to the data obtained for the corresponding control. *, *P* = 0.0494 (unstimulated vs insulin-stimulated WT, *Myog*), *P* = 0.0286 (unstimulated vs insulin-stimulated Onc, *Myog*), *P* = 0.0490 (unstimulated Onc vs WT, *Myog*), *P* = 0.0343 (insulin-stimulated Onc vs WT, *Myog*), *P* = 0.0151 (unstimulated Onc vs WT, *Myh7*), *P* = 0.0117 (unstimulated vs insulin-stimulated Onc and Onc+E200A vs WT, *Myh7*), *P* = 0.0152 (insulin-stimulated Onc+E200A vs WT, *Myh7*), *P* = 0.0137 (unstimulated Onc vs WT, *Myh4*), *P* = 0.0419 (unstimulated Onc+E200A vs WT, *Myh4*), and *P* = 0.0387 (unstimulated Onc+E200A vs Onc, *Myh4*); **, *P* = 0.0021 (Onc+E200A vs WT, *Myog*), *P* = 0.0035 (Onc vs WT, *Myh1*), and *P* = 0.0097 (unstimulated vs insulin-stimulated WT, *Myh7*); ***, *P* = 0.00001 (insulin-stimulated Onc vs WT, *Myh7*) and *P* = 0.0003 (insulin-stimulated Onc+E200A vs Onc, *Myh7*). n = 3 independent experiments.

(d,e) Representative immunofluorescence images (d) and quantification (e) of MHCII positive myotubes in indicated cell lines (left) upon differentiation for 3 days in the presence of either insulin or IGF-1 (top). Data are shown as mean ± SEM. Statistical values were calculated as in c. *, *P* = 0.0333; ***, *P* = 0.0003 (– Ins) and *P* = 0.0002 (+ Ins). n = 2 independent experiments. Scale bar in d, 125 µm.

(f-h) Representative pictures (f) and quantification of the number (g) and girth (h) of the myotubes obtained from the indicated cell lines (left) upon differentiation for the indicated periods of time (top). Data are shown as mean ± SEM. *, *P* = 0.0309 using two-tailed Student's *t* tests. n = 3 independent experiments. Scale bar in f, 100 µm.

Source data for this figure are provided as a Source Data file.

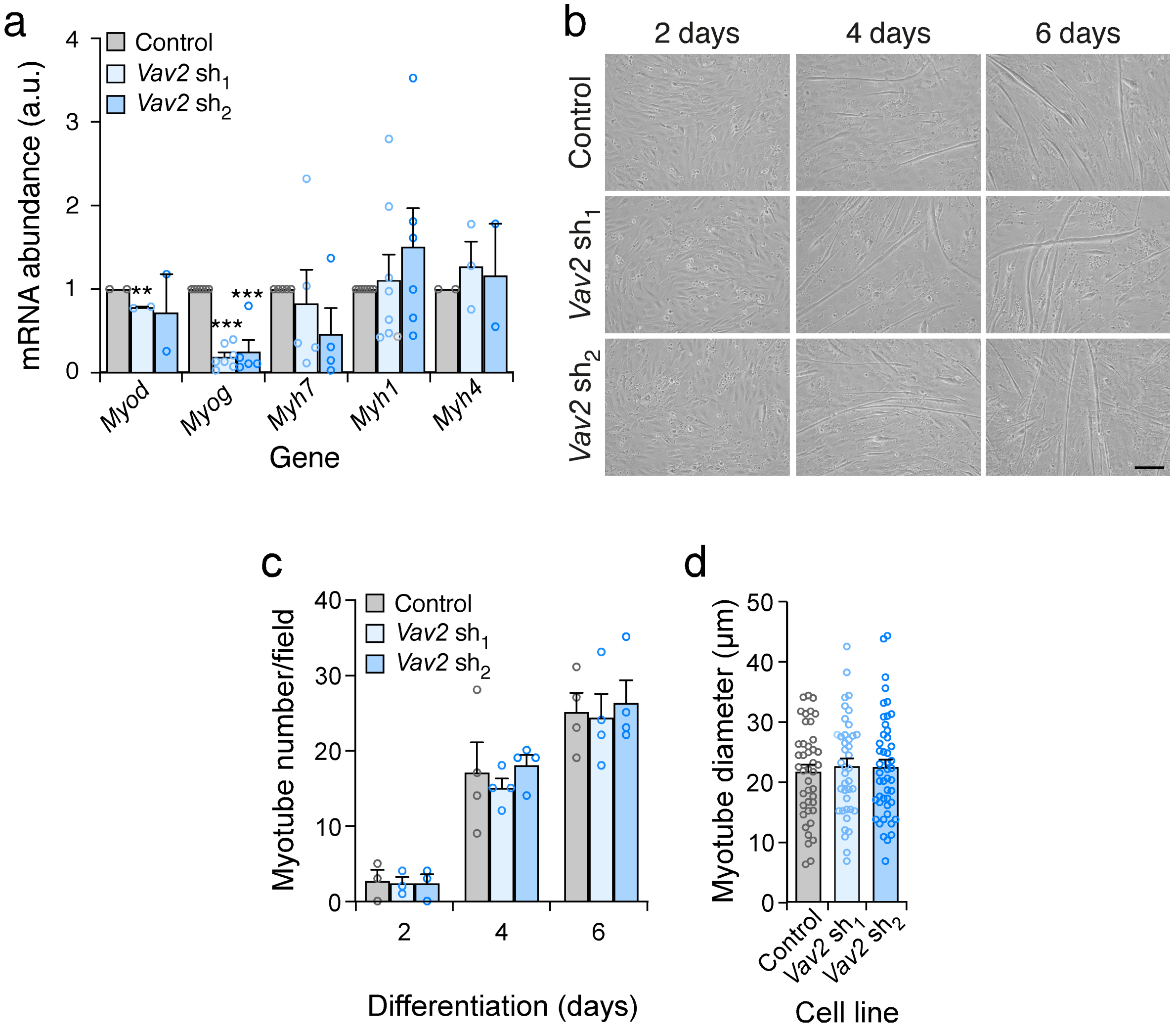

SUPPLEMENTARY FIGURE 6. Effect of the depletion of endogenous Vav2 protein in the differentiation of C2C12 cells

(a) Relative abundance of the indicated transcripts (bottom) upon the differentiation of control (Sc-control) and *Vav2* knockdown (sh_1, sh_2) C2C12 cells for 3 days. Data are shown as mean ± SEM. **, $P = 0.0015$; ***, $P < 0.00001$ (*Vav2* sh_1) and $P = 0.00007$ (*Vav2* sh_2) using two-tailed Student's *t* tests. n = 3 (*MyoD, Myh4*), 4 (*Myh7*) or 5 (*Myog, Myh1*) independent experiments.

(b-d) Representative pictures (b) and quantification of the number (c) and girth (d) of the myotubes obtained from the indicated cell lines (left) upon differentiation for the indicated periods of time (top). Data are shown as mean ± SEM. n = 3 independent experiments. Scale bar in b, 100 µm.

Source data for this figure are provided as a Source Data file.

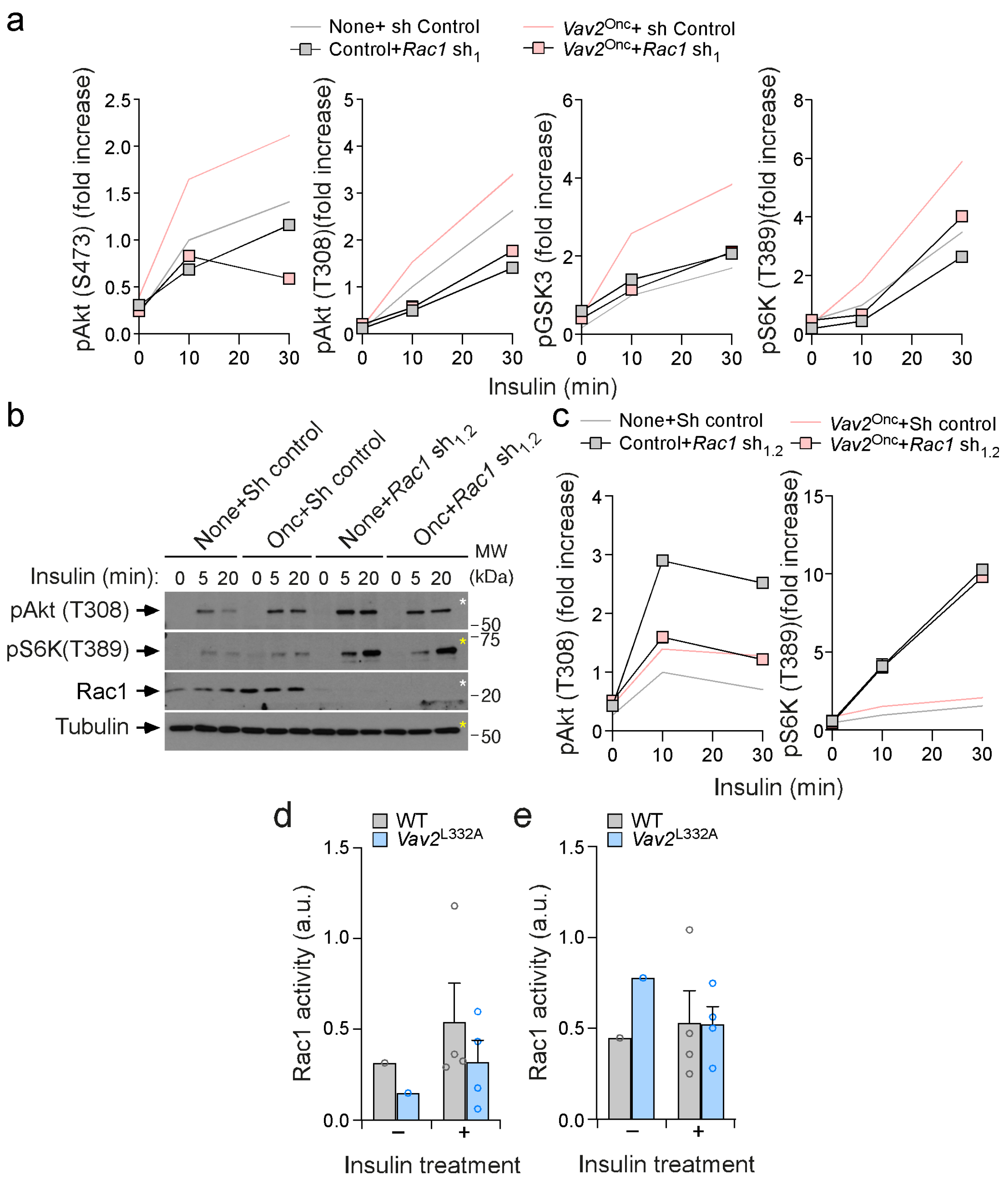

SUPPLEMENTARY FIGURE 7. Vav2 regulates the PI3K–Akt pathway in a Rac-dependent manner

(a) Quantification of the phosphorylation levels of indicated proteins and phosphosites in C2C12 cells (inset) expressing either normal or reduced (but not totally abated) levels (sh_1) of endogenous Rac1 upon stimulation with 75 nM insulin for the indicated period of time (bottom). Data are shown as mean ± SEM. n = 2 independent experiments.

(b) Representative immunoblot showing the phosphorylation level of the indicated proteins (left) in insulin-stimulated control and $Vav2^{Onc}$ cells lines expressing either normal levels of endogenous Rac1 or with the total depletion of endogenous Rac1 protein ($sh_{1.2}$). Cells were stimulated as in a. n = 2 independent experiments.

(c) Quantification of the experiments shown in b. Data are depicted as mean ± SEM. n = 2 independent experiments.

(d,e) Quantification of levels of GTP-bound Rac1 in white adipose tissue (d) and liver (e) of 3-month-old WT and Vav2$^{L332A/L332A}$ mice upon infusion with placebo (–) or an insulin solution (+). Data are shown as mean ± SEM. n = 4 insulin-stimulated animals per condition.

Source data for this figure are provided as a Source Data file.

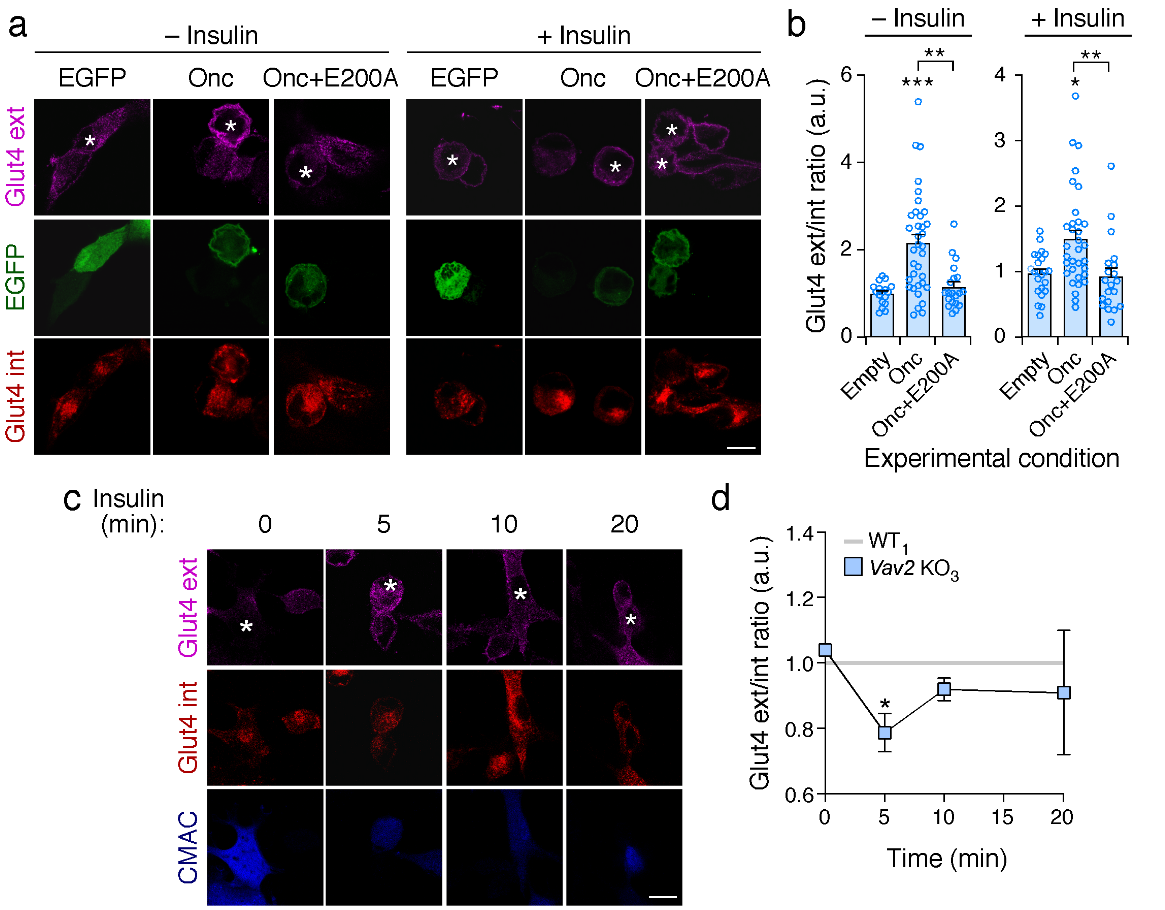

SUPPLEMENTARY FIGURE 8. Effect of Vav2^{Onc} in the translocation of the Glut4 transporter to the plasma membrane

(a,b) Representative fluorescent images (a) and quantification (b) of the translocation of GLUT4 in C2C12 cells stably expressing the bioreporter Myc-Glut4-mCherry upon transfecting the indicated EGFP fusion molecules (top). Determinations were done with or without insulin stimulation as indicated (top). To avoid spurious differences due to the staining, transfected (labelled with an asterisk) and non-transfected cells are shown side by side. Data are shown as mean ± SEM and relative to the values obtained in non-transfected cells in each field (which was given an arbitrary value of 1). *, $P = 0.0293$; **, $P = 0.0011$ (– insulin) and $P = 0.0018$ (+ insulin); ***, $P = 0.0003$ using a Kruskal-Wallis test and two-sided Dunn's multiple comparison tests. n = 3 independent experiments. Ext, externalized (plasma membrane-localized). Int, internal (total Glut). Scale bar in a, 10 µm.

(c) Fluorescence picture of WT_2 (CMAC labelled) and *Vav2* KO_3 cells stably expressing Myc-GLUT4-mCherry treated with insulin for the indicated time period (top). To avoid spurious differences due to the staining, WT cells were stained with CMAC and mixed with a similar amount of Vav2 KO cells before seeding onto the glass. CMAC positive WT cells have been labelled with asterisks. WT_2 refers to clone of C2C12 that had been subjected to the same selection process used to generate *Vav2* KO_3 cells but that it did not undergo the gene-editing process. Scale bar, 10 µm. n = 3 independent experiments.

(d) Quantification of the time-dependent externalization of Glut4 in *Vav2* KO cells versus controls. Data and statistics are as in b. *, $P = 0.0107$ using two-tailed Student's *t* tests. n = 3 independent experiments.

Source data for this figure are provided as a Source Data file.

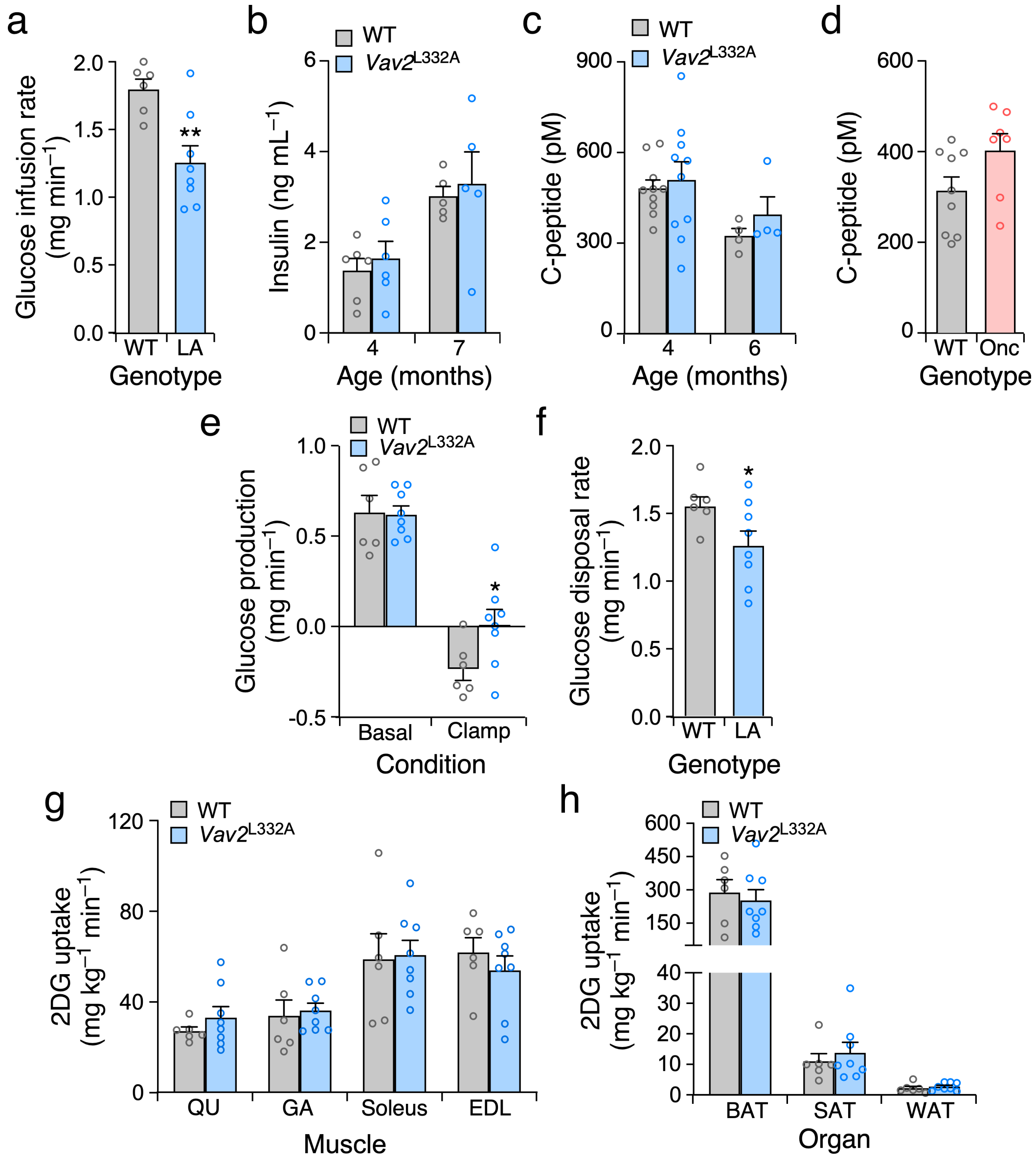

SUPPLEMENTARY FIGURE 9. Evaluation of glucose-related parameters in *Vav2*$^{L332A/L332A}$ mice

(a) Glucose infusion rate at the steady state of the hyperinsulinemic-euglycemic clamp experiments performed on 3-month-old animals of the indicated genotype (bottom). Data are shown as mean ± SEM. **, $P = 0.0031$ using two-tailed Student's t tests with Welch correction. n = 6 (WT) and 8 (*Vav2*$^{L332A/L332A}$) mice. LA, *Vav2*$^{L332A/L332A}$.

(b) Concentration of insulin in the plasma of animals of the indicated genotypes (inset) and ages (bottom) that were fasted for 6 hours. Data represent the mean ± SEM. n = 6 (4-month-old) or 5 (7-month-old) animals per group.

(c) Concentration of the C-peptide in the plasma of fasted animals of the indicated genotypes (inset) and ages (bottom) 15 min after being injected with a bolus of glucose. Data represent the mean ± SEM. n = 10 (4-month-old) and 4 (6-month-old) animals per group.

(d) Concentration of the C-peptide in the plasma of fasted 6-month-old animals of indicated genotypes (bottom) 15 min after being injected with a bolus of glucose. Data represent the mean ± SEM. n = 9 (WT) and 7 (*Vav2*$^{Onc/Onc}$) mice.

(e) Hepatic glucose production by 3-month-old animals of indicated genotypes at basal and the hyperinsulinemic (Clamp) conditions (bottom). Data are shown as mean ± SEM. *, $P = 0.0360$ using two-tailed Student's t tests with Welch correction. n = 6 (WT) and 8 (*Vav2*$^{L332A/L332A}$) mice.

(f) Glucose disposal by 3-month-old animals of indicated genotypes. Data are shown as mean ± SEM. *, $P = 0.0444$ using two-tailed Student's t tests with Welch correction. n = 6 (WT) and 8 (*Vav2*$^{L332A/L332A}$) mice.

(g,h) Glucose uptake in peripheral tissues in 3-month-old animals of indicated genotypes. Data are shown as mean ± SEM. n = 6 (WT) and 8 (*Vav2*$^{L332A/L332A}$) mice. 2DG, 2-deoxyglucose; QU, quadriceps; GA, gastrocnemius; EDL, extensor digitorum longus; BAT, brown adipose tissue; SAT, subcutaneous adipose tissue; WAT, white adipose tissue.

Source data for this figure are provided as a Source Data file.

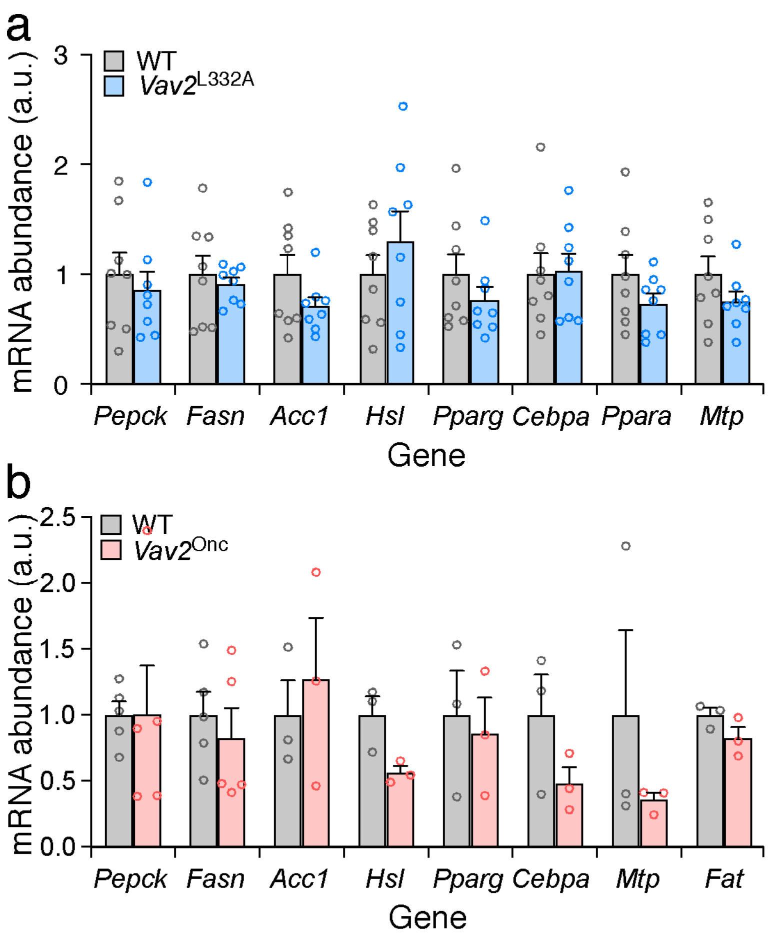

SUPPLEMENTARY FIGURE 10. Effect of deregulated Vav2 catalytic output in the expression levels of mRNAs for metabolic enzymes in white adipose tissue

(a,b) mRNA abundance of the indicated transcripts in WATs from 4-month-old *Vav2*$^{L332A/L332A}$ (a) and *Vav2*$^{Onc/Onc}$ (b) mice. Data are shown as mean ± SEM. n = 8 (a) and 3 to 5 (b) mice per group.

Source data for this figure are provided as a Source Data file.

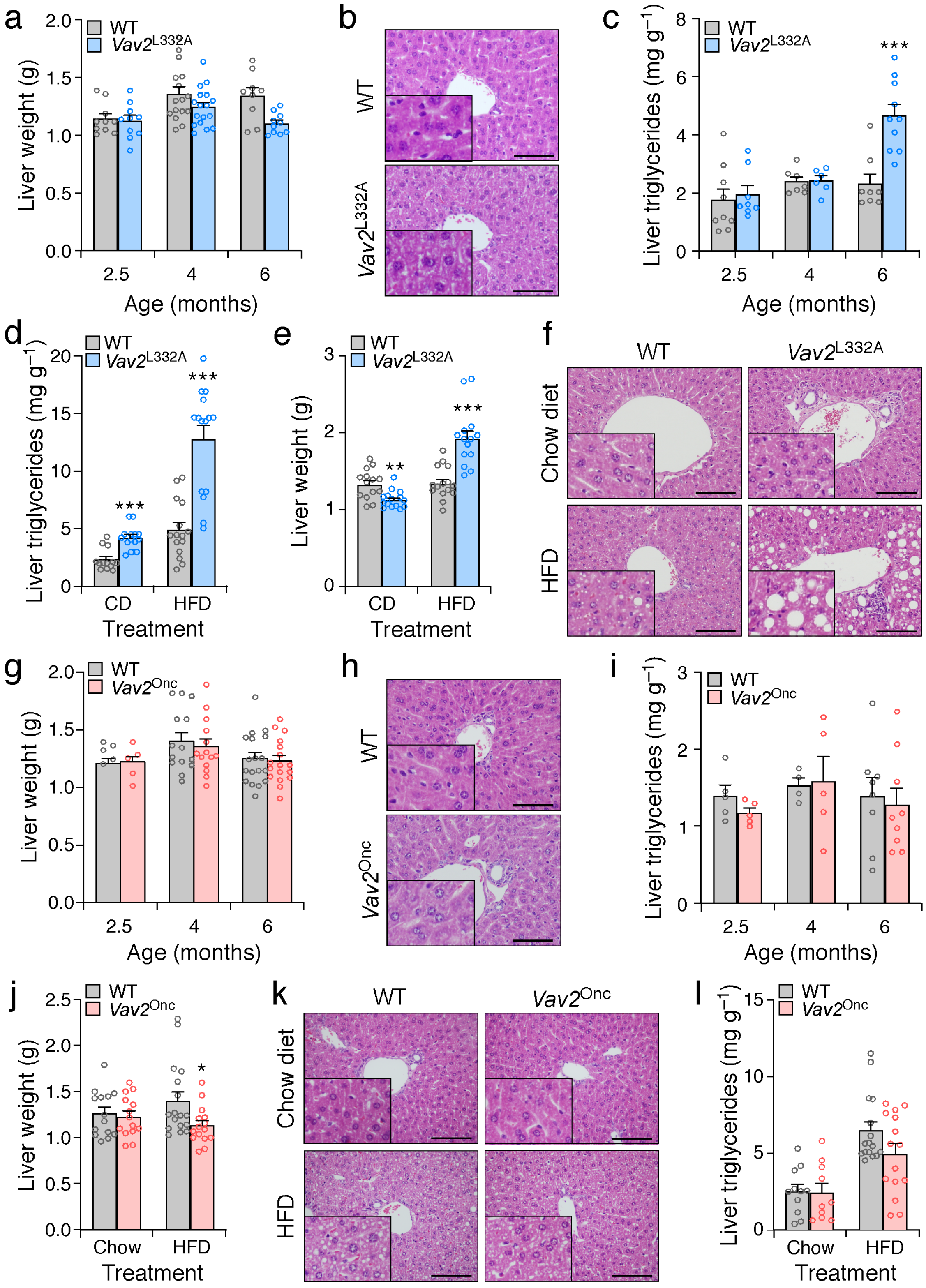

SUPPLEMENTARY FIGURE 11. The Vav2 catalytic output mildly influences liver metabolism

(a) Weight of livers from CD-fed animals of the indicated genotypes (inset) and ages (bottom). Data are presented as mean ± SEM. n = 10 (2.5-month-old animals and 6-month-old *Vav2*$^{L332A/L332A}$ mice), 16 (4-month-old WT mice), 17 (4-month-old *Vav2*$^{L332A/L332A}$ mice) and 9 (6-month-old WT animals).

(b) Representative images of liver sections from CD-fed 6-month-old mice of the indicated genotypes (left). Scale bar, 100 μm. n = 10 mice per group.

(c) Triglyceride content in livers from mice of the indicated genotypes (inset) and ages (bottom). Data are shown as mean ± SEM. ***, *P* = 0.0004 using two-tailed Student's *t* test. n = 9 (2.5-month-old WT animals), 8 (2-month-old *Vav2*$^{L332A/L332A}$ and 6-month-old WT mice), 7 (4-month-old WT mice), 6 (4-month-old *Vav2*$^{L332A/L332A}$ mice).

(d) Triglyceride content in livers from CD- or HFD-fed (bottom) 6-month-old animals of the indicated genotypes (inset). Data are presented as mean ± SEM. ***, *P* = 0.000029 (CD) and *P* = 0.000003 (HFD) using two-tailed Student's *t* test. n = 13 (CD-fed WT), 14 (CD-fed *Vav2*$^{L332A/L332A}$) and 15 (HFD-fed) animals.

(e) Weight of livers from CD- and HFD-fed animals (bottom) of the indicated genotypes (inset). Data are presented as mean ± SEM. **, *P* = 0.0016; ***, *P* = 0.000016 using two-tailed Student's *t* test. n = 14 (CD-fed WT and HFD-fed *Vav2*$^{Onc/Onc}$) and 15 (HFD-fed WT and CD-fed *Vav2*$^{Onc/Onc}$) animals per group.

(f) Representative images of liver sections from either CD- or HFD-fed (left) 6-month-old mice of the indicated genotypes (top). Scale bar, 100 μm. n= 15 animals per group.

(g) Weight of livers from CD-fed animals of the indicated genotypes (inset) and ages (bottom). Data are presented as mean ± SEM. n = 5 (2.5-month-old animals), 14 (4-month-old mice), 18 (6-month-old WT mice) and 17 (6-month-old *Vav2*$^{Onc/Onc}$ animals).

(h) Representative liver sections from 6-month-old mice of the indicated genotypes (left) that were maintained under CD conditions. Scale bar, 100 μm. n = 8 (WT) and 9 (*Vav2*$^{Onc/Onc}$) animals.

(i) Liver triglyceride content in mice of the indicated genotypes (inset) and ages (bottom). Data represent the mean ± SEM. n = 5 (2.5-month-old animals and 4-month-old *Vav2*$^{Onc/Onc}$ animals), 4 (4-month-old WT mice), 8 (6-month-old WT mice) and 9 (6-month-old *Vav2*$^{Onc/Onc}$ animals).

(j) Weight of livers from CD- and HFD-fed animals (bottom) of the indicated genotypes (inset) and ages (bottom). Data are presented as mean ± SEM. *, *P* = 0.0251 using two-tailed Student's *t* test. n = 14 (CD-fed), 15 (HFD-fed *Vav2*$^{Onc/Onc}$) and 17 (HFD-fed WT) animals per group.

(k) Representative images of liver sections from CD- and HFD-fed (left) 6-month-old mice of the indicated genotypes (top). Scale bar, 100 μm. n = 10 (CD-fed *Vav2*$^{Onc/Onc}$), 11 (CD-fed WT), 15 (HFD-fed *Vav2*$^{Onc/Onc}$) and 16 (CD-fed WT) animals per group.

(l) Triglyceride content in livers from CD- or HFD-fed (bottom) 6-month-old animals of the indicated genotypes (inset). Data are presented as mean ± SEM. n = 10 (CD-fed *Vav2*$^{Onc/Onc}$), 11 (CD-fed WT), 15 (HFD-fed *Vav2*$^{Onc/Onc}$) and 16 (CD-fed WT) animals per group.

Source data for this figure are provided as a Source Data file.

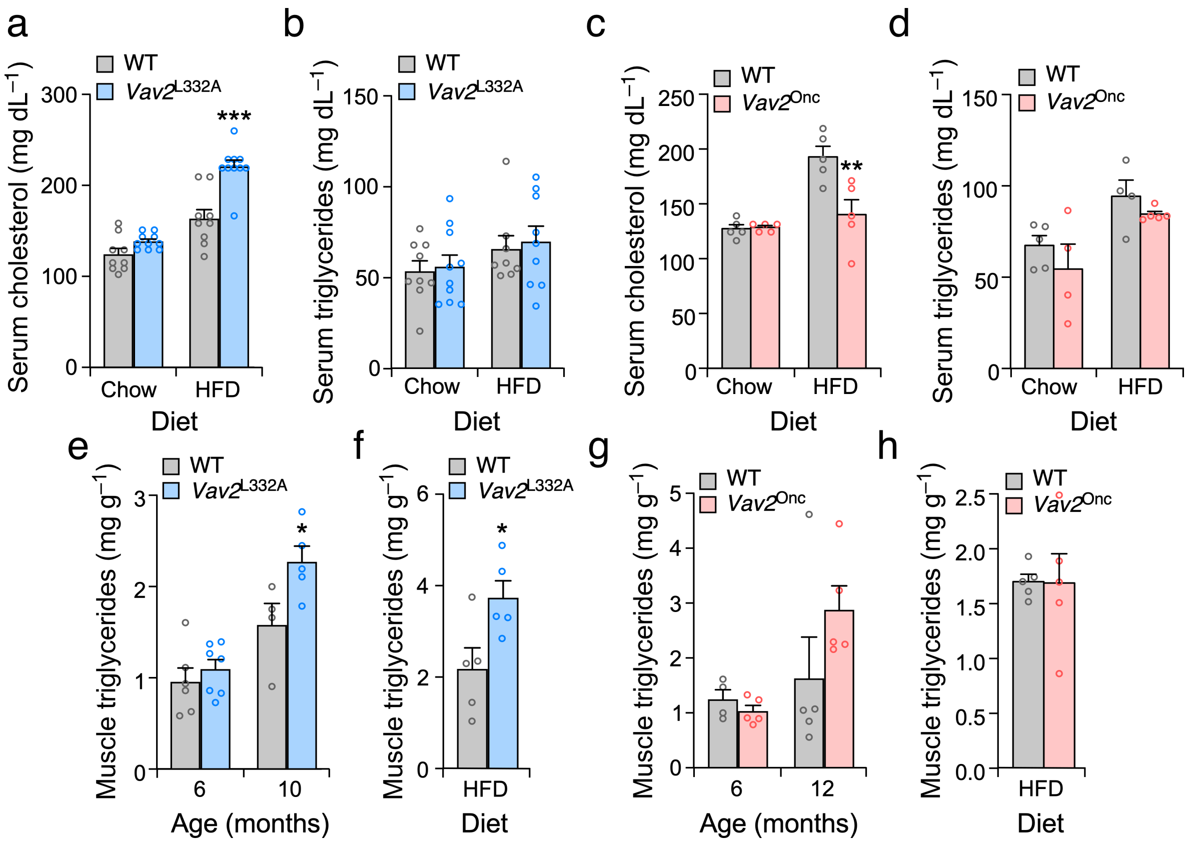

SUPPLEMENTARY FIGURE 12. Evaluation of cholesterol and triglyceride content in mice with deregulated Vav2 catalytic output

(a,b) Serum cholesterol (a) and triglyceride (b) levels in 6-month-old animals of indicated genotypes (insets) that were maintained under the specified diet conditions (bottom). Data are shown as mean ± SEM. ***, *P* = 0.0001 using two-way ANOVA and Holm-Sidak's multiple comparison tests. n = 8 (HFD-fed WT mice in b) 9 (WT animals in a, CD-fed WT and HFD-fed *Vav2*$^{L332A/L332A}$ mice in b) and 10 (*Vav2*$^{L332A/L332A}$ mice in a, CD-fed *Vav2*$^{L332A/L332A}$ mice in b) mice per condition.

(c,d) Serum cholesterol (c) and triglycerides (d) in 6-month-old mice of indicated genotypes (top) and that were fed with chow and high fat diets as indicated (bottom). Data are shown as mean ± SEM. **, *P* = 0.001 using two-way ANOVA and Holm-Sidak's multiple comparison tests. n = 5 mice per group.

(e-h) Intramuscular triglyceride levels in the gastrocnemius of animals of indicated genotypes (top) and ages (bottom) that were fed with chow (e,g) or high fat diet. In the case of panels f and h, 6-month-old animals were used in the experiments. Data are shown as mean ± SEM. *, *P* = 0.0312 (f) and 0.0457 (e) using two-tailed Student's *t* tests. n = 6 (6-month-old WT mice, e), 4 (10-month-old WT mice, e and 6-month-old WT mice, g), 7 (6-month-old *Vav2*$^{L332A/L332A}$ mice, e) and 5 (f, g, h plus the 6-month-old *Vav2*$^{L332A/L332A}$ mice in e).

Source data for this figure are provided as a Source Data file.

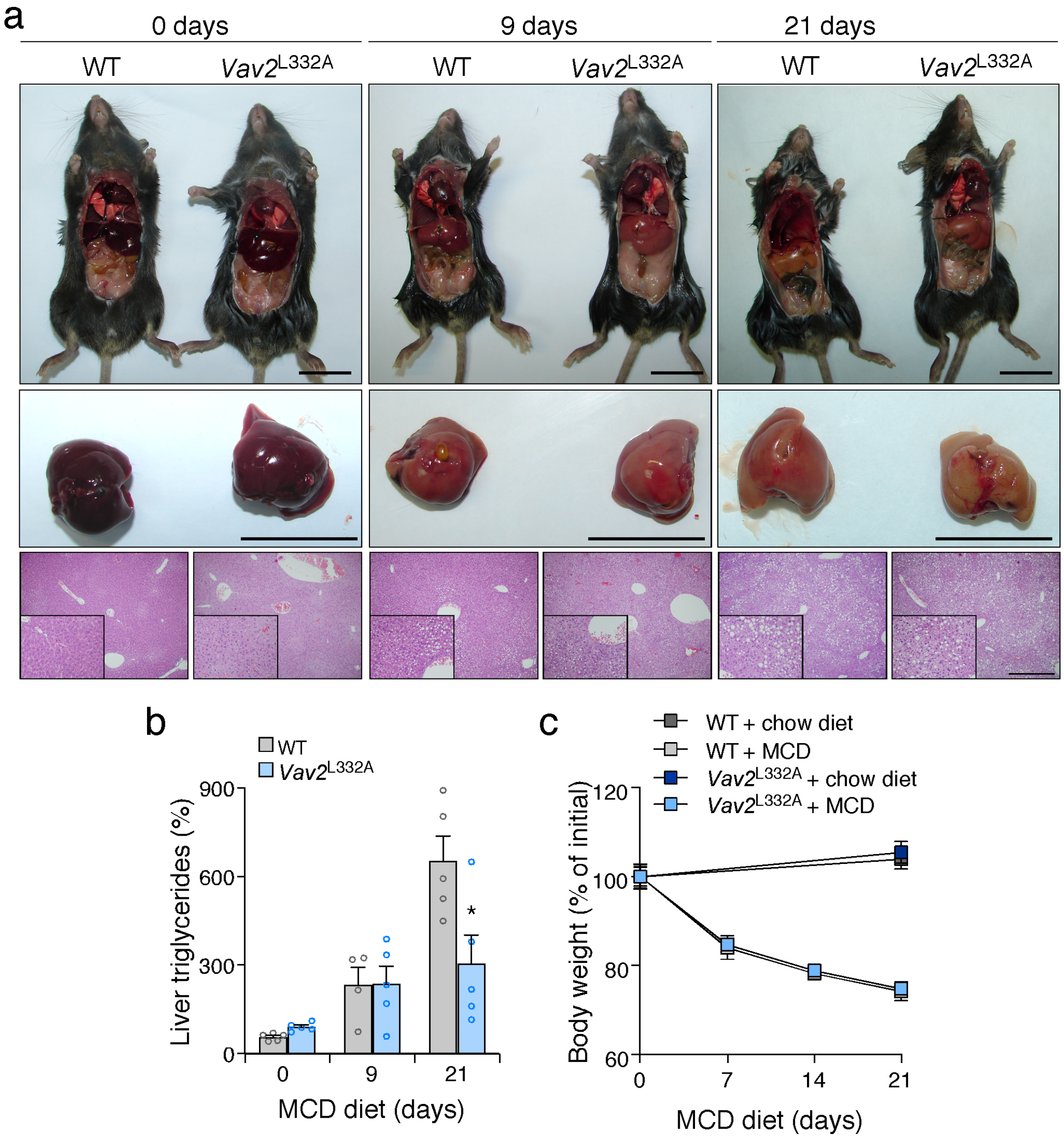

SUPPLEMENTARY FIGURE 13. *Vav2*$^{L332A/L332A}$ mice behave as the WT counterparts when subjected to a methionine- and choline-deficient diet

(a) Representative pictures of the animals of indicated genotypes(top panels) and liver removed from them (middle and bottom panels) that were maintained under a methionine choline deficient diet (MCD) for the indicated periods of time (top). Scale bar, 2 cm (top and middle panels) and 400 µm (bottom panels). n = 4 (WT mice, 9th day treatment time point) and 5 (rest of time points) animals per group.

(b) Accumulation of triglycerides in the liver of mice fed with a MCD for the indicated periods of time. Data are shown as mean ± SEM. *, P = 0.0267 using two-tailed Student's t tests. n = 4 (WT, 9th day treatment time point) and 5 (rest of time points) mice per group.

(c) Evolution of the body weight of animals of indicated genotypes maintained under either chow or MCD diets for the indicated periods of time. Data are shown as mean ± SEM. n = 6 (chow diet), 5 (14th and 21st day treatment time points), 9 (*Vav2*$^{L332A/L332A}$, 7th day treatment time point) and 10 (rest) animals.

Source data for this figure are provided as a Source Data file.

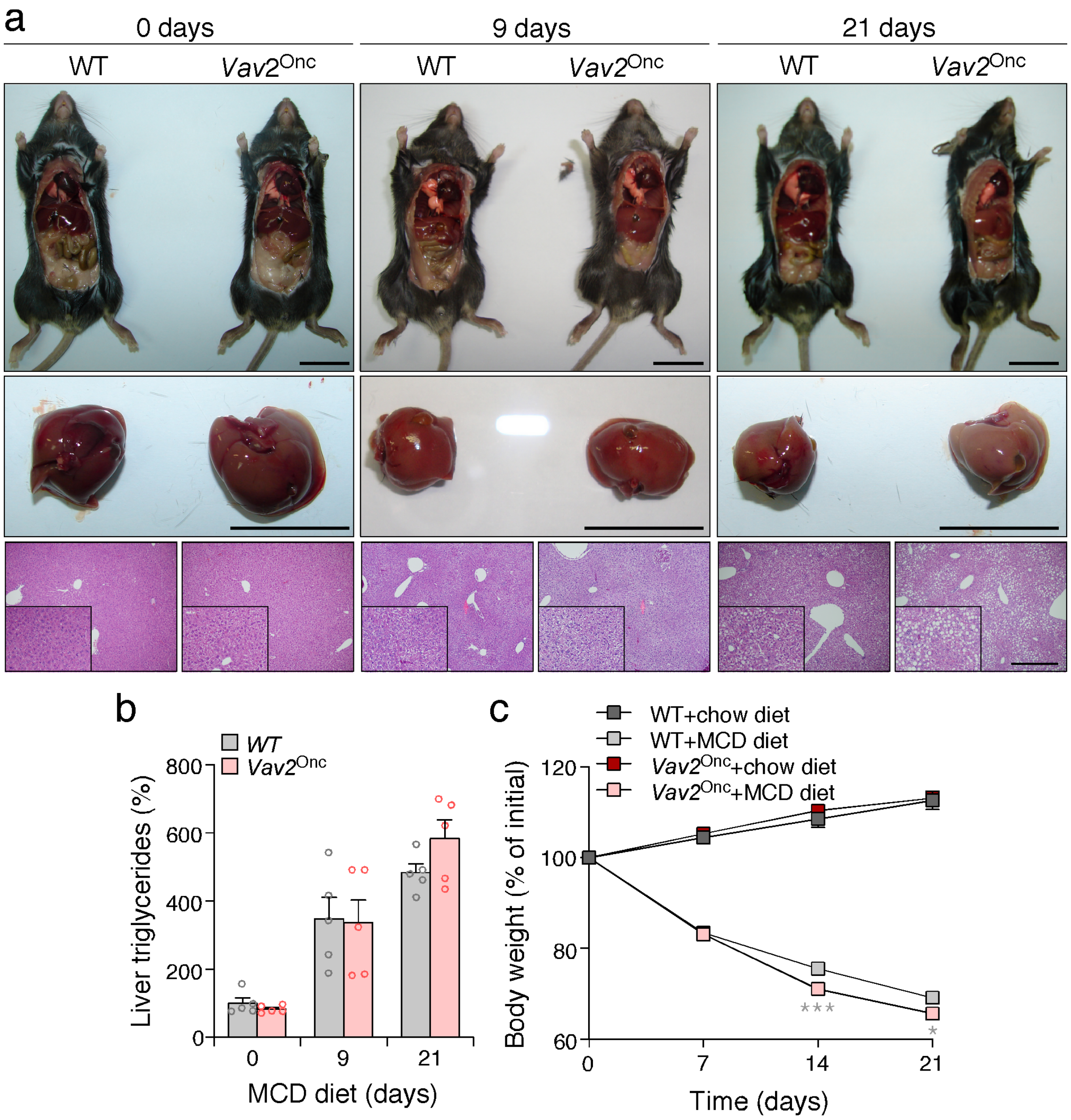

SUPPLEMENTARY FIGURE 14. *Vav2*$^{Onc/Onc}$ mice behave as the WT counterparts when subjected to a methionine- and choline-deficient diet

(a) Representative pictures of animals of indicated genotypes (top panels) and the liver removed from each of them (middle and bottom panels) of the indicated genotypes that were maintained under a MCD for the indicated periods of time (top). Scale bar, 2 cm (top and middle panels) and 400 µm (bottom panels). n = 5 animals per group.

(b) Accumulation of triglycerides in the liver of mice fed with a MCD for the indicated periods of time. Data are shown as mean ± SEM (n = 5 animals per group).

(c) Evolution of the body weight of animals of the indicated genotype that were fed with either a chow diet or a MCD for the indicated periods of time. Data are shown as mean ± SEM. *, $P = 0.019$; ***, $P = 0.00099$ using two-tailed Student's *t* tests. n = 5 animals per group.

Source data for this figure are provided as a Source Data file.

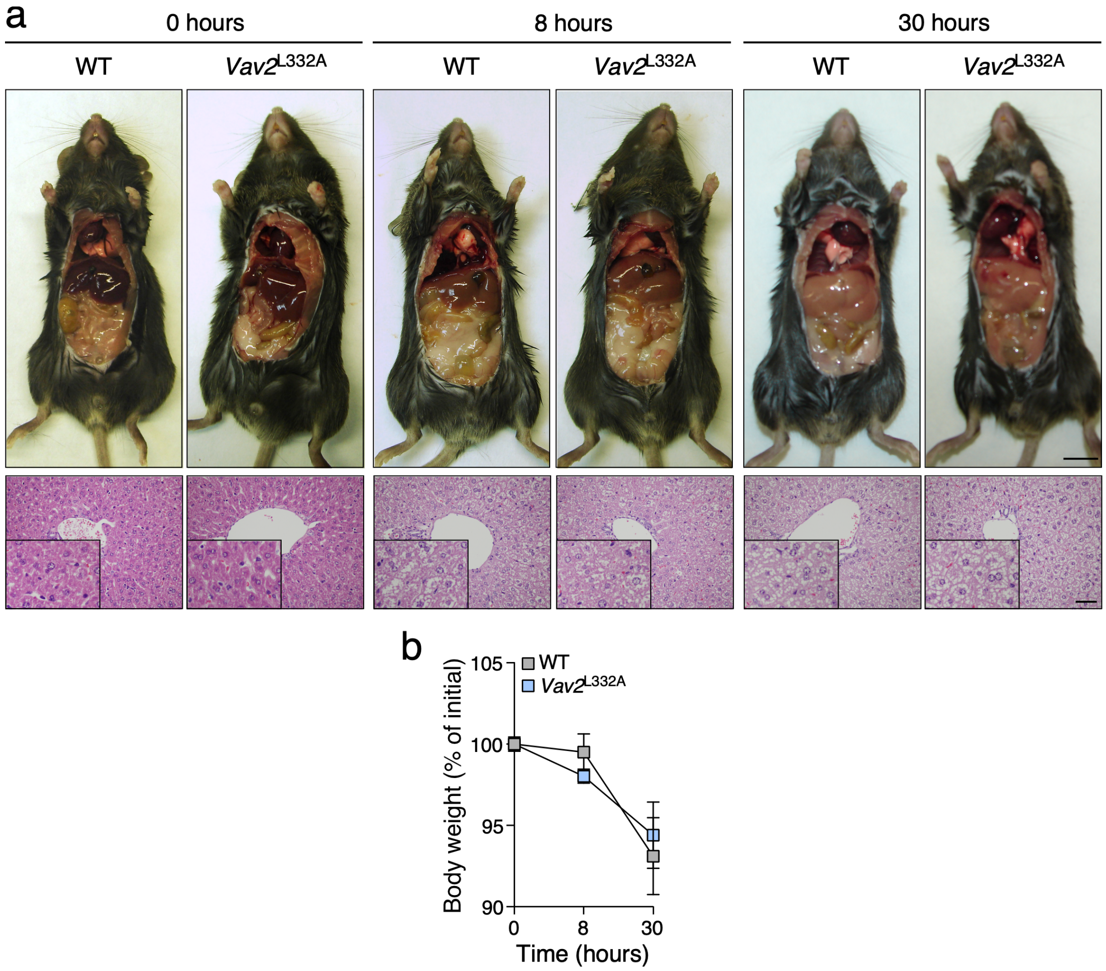

SUPPLEMENTARY FIGURE 15. *Vav2*$^{L332A/L332A}$ mice behave as WT counterparts when treated with tunicamycin

(a) Representative pictures of animals of indicated genotypes (top panels) and the liver removed from each of them (middle and bottom panels) of the indicated genotypes after being treated intraperitoneally with tunicamycin for the indicated periods of time. Scale bars, 1 cm (animals) and 50 µm (liver sections). n = 3 animals per group.

(b) Evolution of the body weight of animals of indicated genotypes during the time of the tunicamycin administration. Data are shown as mean ± SEM. n = 3 animals per group.

Source data for this figure are provided as a Source Data file.

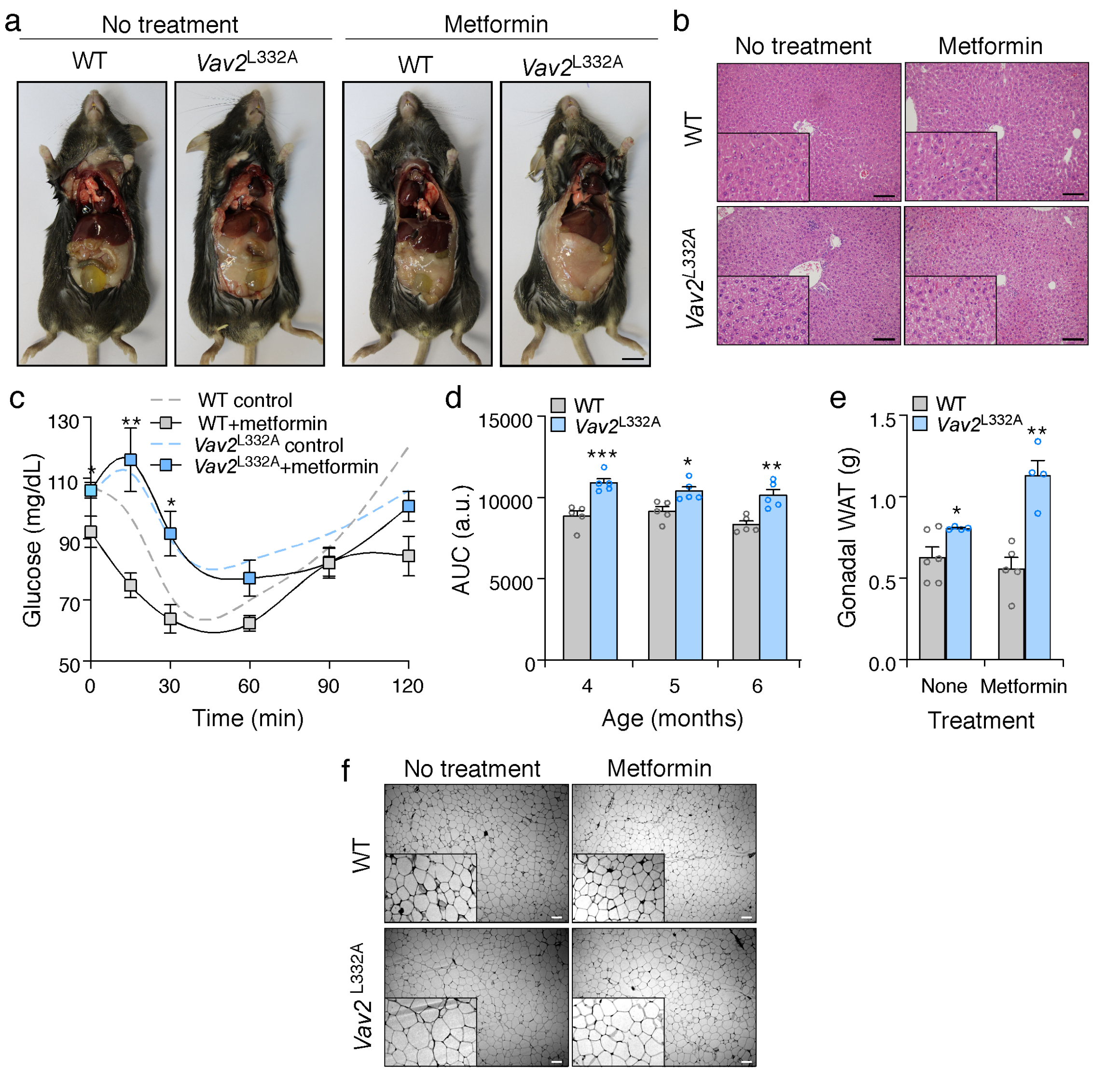

SUPPLEMENTARY FIGURE 16. Metformin does not block the metabolic dysfunctions present in *Vav2*$^{L332A/L332A}$ mice

(a) Representative pictures of 6-month-old mice of indicated genotypes that were treated with metformin for 4 months. Scale bar, 1 cm.

(b) Representative images of liver sections from 6-month-old mice of indicated genotypes (left) that were either left untreated or treated with metformin (top) for four months. Scale bar, 100 µm. n = 5 animals per group.

(c) Response of 4-month-old animals maintained with or without metformin to an insulin tolerance test. Data are shown as mean ± SEM. *, $P = 0.0453$ (0 minutes time point) and $P = 0.01238$ (30 minutes time point); **, $P = 0.0062$ using two-tailed Student's *t* tests. n = 5 animals per group.

(d) Area under the curve (AUC) responses of metformin-treated mice of indicated genotypes (inset) and ages (bottom) to an infusion of insulin. Data are shown as mean ± SEM. *, $P = 0.0119$; **, $P = 0.0025$; ***, $P = 0.0009$ using two-tailed Student's *t* tests. n = 5 animals per group.

(e,f) Weight (e) and representative image (f) of the gonadal WAT mass present in 6-month-old animals of the indicated genotypes (inset) that were maintained either in the absence or presence of metformin as specified (bottom). Data are presented as mean ± SEM. *, $P = 0.0361$; **, $P = 0.0012$ relative to WT controls using two-tailed Student's *t* tests (n = 5 animals per experimental group). Scale bar, 100 µm.

Source data for this figure are provided as a Source Data file.

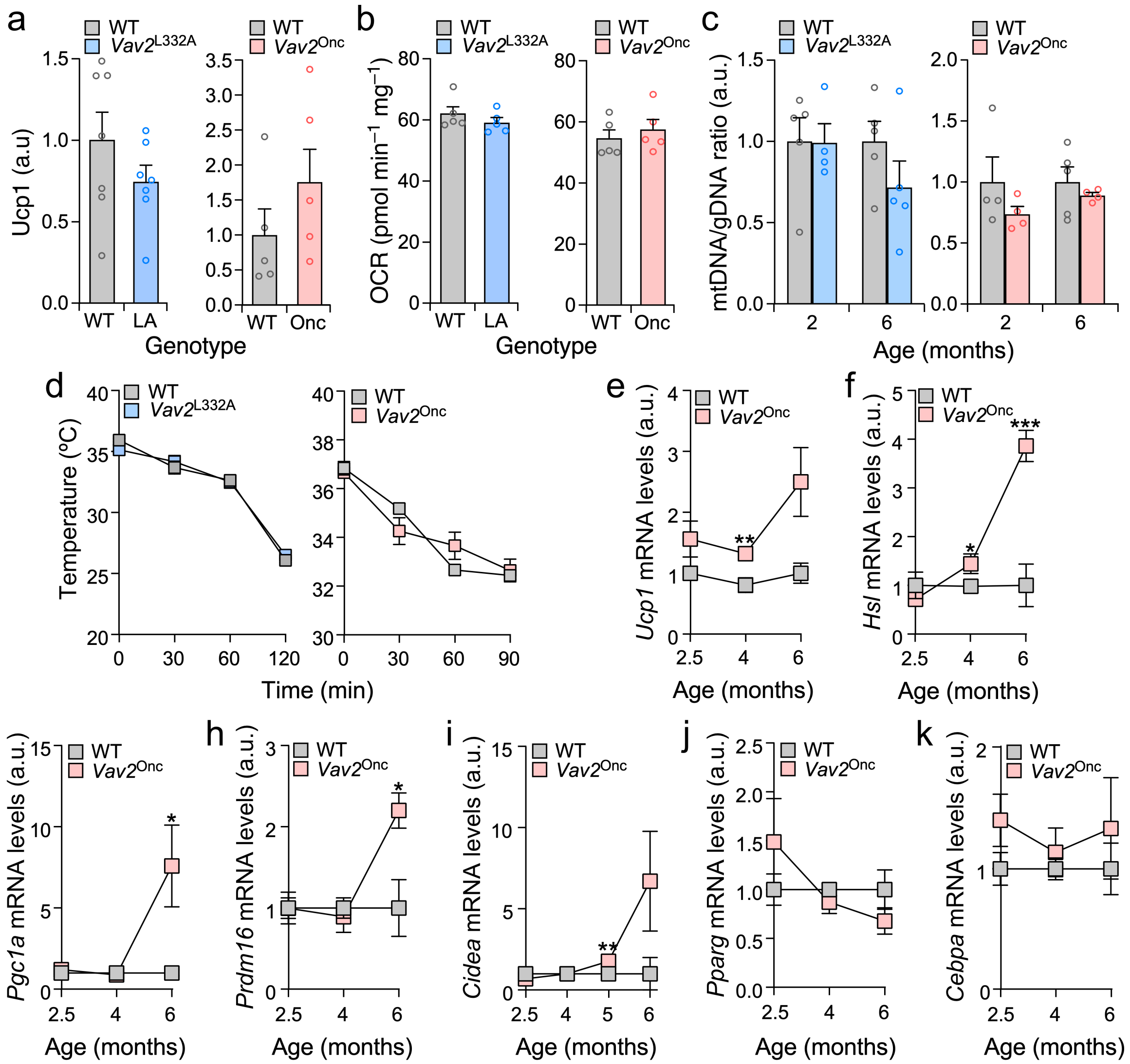

SUPPLEMENTARY FIGURE 17. Evaluation of BAT metabolic parameters in mice with deregulated Vav2 catalytic output

(a) Quantification of Ucp1 protein levels in BAT extracts from 4-month-old mice of indicated genotypes. Tubulin was used as loading control. Data are shown as mean ± SEM. n = 7 (in the *Vav2*$^{L332A/L332A}$ experiments) and 5 (in the *Vav2*$^{Onc/Onc}$ experiments) animals per group.

(b) Basal oxygen consumption rate (OCR) of BAT explants from 3.5-month-old animals of indicated genotypes. Data are presented as mean ± SEM. n = 5 mice per group.

(c) Ratio of mitochondrial (mtDNA) to genomic (gDNA) DNA in BAT samples from mice of indicated genotypes and ages (bottom). n = 4 (2-month-old *Vav2*$^{L332A/L332A}$, 2-month-old and 6-month-old *Vav2*$^{Onc/Onc}$ in the right panel) and 5 (rest) mice per group.

(d) Rectal temperature of 6-month-old mice of indicated genotypes (inset) during cold exposure for the indicated periods of time (bottom). Data are presented as mean ± SEM. n = 6 (WT), 3 (*Vav2*$^{L332A/L332A}$) and 5 (right panel) mice per group.

(e-k) Expression levels of indicated genes in the BAT from mice of indicated genotypes (inset) and ages (bottom). Data are shown as mean ± SEM. *, P = 0.0328 (f), P = 0.0309 (g), and P = 0.0145 (h); **, P = 0.0068 (e) and, P = 0.0361 (i); ***, P = 0.0009 using two-tailed Student's t tests. n = 5 (2-month-old in e, f, h and k, 6-month-old in g and i, 6-month-old WT in e, 2-month-old WT in j and 6-month-old *Vav2*$^{Onc/Onc}$ in f, j and k), 8 (5-month-old WT, i), 6 (4- and 6-month-old *Vav2*$^{Onc/Onc}$, h), 7 (6-month-old *Vav2*$^{Onc/Onc}$ in e and 5-month-old

Vav2$^{Onc/Onc}$ in i), 9 (4-month-old WT, e), 12 (4-month-old WT, f), 10 (4-month-old *Vav2*$^{Onc/Onc}$, e and f), 3 (6-month-old WT in j) and 4 (rest) mice per genotype and age group.

Source data for this figure are provided as a Source Data file.

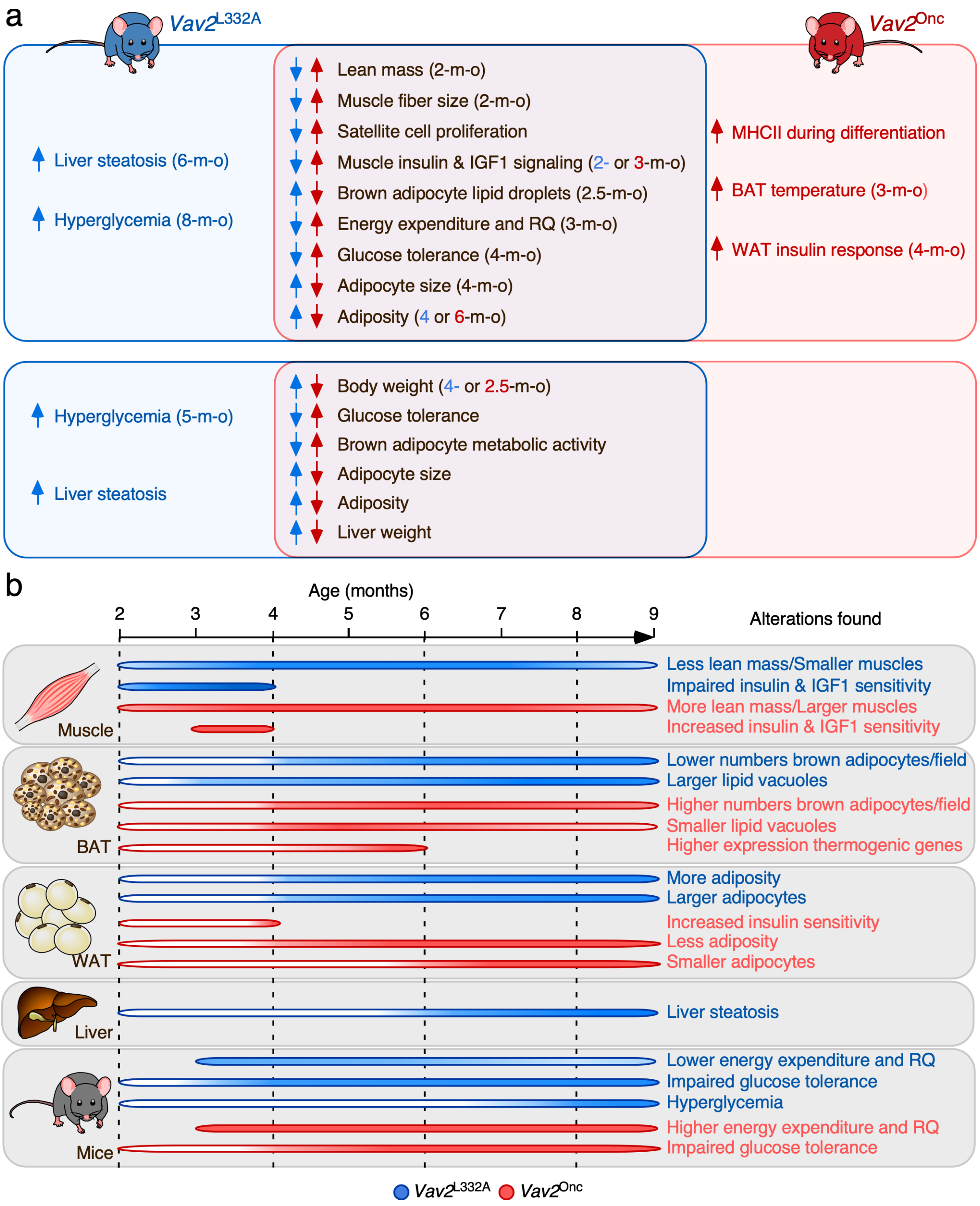

SUPPLEMENTARY FIGURE 18. Summary of the time-dependent evolution of the alterations in metabolic-related tissues found in mice with deregulated Vav2 catalytic activity

(a) Summary of the genotype-specific and overlapping phenotypes found in *Vav2*$^{L332A/L332A}$ (blue) and *Vav2*$^{Onc/Onc}$ (red) mice.

(b) Evolution of changes in skeletal muscle, BAT, WAT, liver, and the whole mouse exhibited by *Vav2*$^{L332A/L332A}$ and *Vav2*$^{Onc/Onc}$ mice. The time frame during which the indicated parameters have been monitored are indicated by rounded rectangles. The time frame in which a specific defect has been detected is indicated by gradients of blue (in the case of *Vav2*$^{L332A/L332A}$ mice) and red (in the case of *Vav2*$^{Onc/Onc}$ mice) color.

SUPPLEMENTARY TABLE 1. qRT-PCR-determined levels of indicated transcripts (left) in tissues from WT, *Vav2*$^{L332A/L332A}$ (LA) and *Vav2*$^{Onc/Onc}$ (Onc) mice. *n* = 3.

		Pancreas	Muscle	Liver	WAT	BAT
Vav2	WT	1.00 ± 0.09	0.35 ± 0.06	493.64 ± 88.83	63.59 ± 8.59	316.08 ± 20.74
	LA	1.02 ± 0.05	0.39 ± 0.09	389.84 ± 74.75	63.87 ± 20.23	300.54 ± 20.23
Rac1	WT	1.00 ± 0.12	2.68 ± 0.31	95.39 ± 17.37	381.75 ± 34.49	37.43 ± 3.91
	LA	0.90 ± 0.14	1.56± 0.40	96.37 ± 18.42	489.04± 69.70	36.98± 4.93
Prex1	WT	1.00 ± 0.22	2.93 ± 0.22	20.97 ± 2.19	43.79 ± 5.71	2.18 ± 0.22
	LA	0.96 ± 0.18	2.91 ± 0.15	18.19 ± 3.33	47.43 ± 9.01	1.82 ± 0.29
Prex2	WT	1.00 ± 0.13	147.04 ± 22.47	741.27 ± 133.07	10640.6 ± 484.00	348.84 ± 57.17
	LA	0.93 ± 0.02	151.70 ± 49.14	589.74 ± 25.17	8492.82 ± 711.09	283.66 ± 56.88
Tiam1	WT	1.00 ± 0.07	0.16 ± 0.02	0.14 ± 0.03	0.68 ± 0.16	0.03 ± 0.00
	LA	0.75 ± 0.02	0.23 ± 0.03	0.16 ± 0.01	0.73 ± 0.17	0.03 ± 0.01
Vav3	WT	1.00 ± 0.03	0.18 ± 0.05	0.03 ± 0.00	10.53 ± 1.34	0.96 ± 0.10
	LA	1.04 ± 0.17	0.30 ± 0.04	0.02 ± 0.00	11.03 ± 0.41	0.98 ± 0.21

		Pancreas	Muscle	Liver	WAT	BAT
Vav2	WT	1.00 ± 0.21	0.17 ± 0.01	154.24 ± 40.69	18.91 ± 1.78	35.85 ± 3.45
	Onc	0.76 ± 0.05	0.15 ± 0.03	107.97 ± 27.66	21.01 ± 2.03	35.68 ± 4.96
Rac1	WT	1.00 ± 0.06	1.00 ± 0.06	50.45 ± 17.68	203.41 ± 45.83	8.01 ± 1.24
	Onc	1.06 ± 0.05	0.92 ± 0.28	40.17 ± 7.68	326.86 ± 74.24	10.28 ± 0.56
Prex1	WT	1.00 ± 0.10	0.46 ± 0.09	13.57 ± 1.53	46.79 ± 10.67	1.10 ± 0.12
	Onc	0.80 ± 0.04	0.36 ± 0.03	13.29 ± 1.23	66.35 ± 17.21	1.49 ± 0.19
Prex2	WT	1.00 ± 0.09	22.61 ± 2.59	171.30 ± 16.28	2206.87 ± 783.70	46.72 ± 6.35
	Onc	0.90 ± 0.13	18.49 ± 3.97	229.81 ± 36.50	2179.89 ± 601.65	47.58 ± 5.02
Tiam1	WT	1.00 ± 0.18	0.07 ± 0.01	0.11 ± 0.01	0.56 ± 0.04	0.01 ± 0.00
	Onc	1.01 ± 0.16	0.06 ± 0.01	0.13 ± 0.04	0.54 ± 0.07	0.01 ± 0.00
Vav3	WT	1.00 ± 0.24	0.10 ± 0.02	0.01 ± 0.00	16.87 ± 2.00	0.47 ± 0.01
	Onc	1.14 ± 0.15	0.08 ± 0.02	0.01 ± 0.00	23.71 ± 1.67	0.58 ± 0.06

SUPPLEMENTARY TABLE 2. Metabolic parameters of *Vav2*$^{L332A/L332A}$ and *Vav2*$^{Onc/Onc}$ mice. The indicated parameters (left) were measured in individually caged CD-fed mice of the indicated ages (top) and genotypes (top). Data are shown as mean ± SEM. Statistically significant values are shown in bold. *, $P < 0.05$; **, $P < 0.01$ using two-tailed Student's *t* tests. n = 6 (5-month old animals), 12 (3-month-old animals), 5 (10-month-old animals and 12-month-old WT mice) and 7 (12-month-old *Vav2*$^{L332A/L332A}$ mice). Source data for this figure are provided as a Source Data file.

		**5-month-old**		**12-month-old**		**3-month-old**		**10-month-old**		**12-month-old**	
		**WT**	***Vav2*^{L332A}**	**WT**	***Vav2*^{L332A}**	**WT**	***Vav2*^{Onc}**	**WT**	***Vav2*^{Onc}**	**WT**	***Vav2*^{Onc}**
**Food intake (g)**		7.68 ± 0.25	6.93 ± 0.63	10.15 ± 0.82	**13.63 ± 0.73***	8.22 ± 0.35	**9.40 ± 0.68***	9.60 ± 0.72	9.56 ± 0.15	7.34± 0.51	6.06 ± 0.31
**Food intake/lean mass**		0.27 ± 0.01	0.26 ± 0.02	0.34 ± 0.03	**0.49 ± 0.02****	0.50 ± 0.02	0.51 ± 0.02	0.38 ± 0.02	0.35 ± 0.01	0.29± 0.01	**0.23± 0.01***
**Rectal temperature (ºC)**		38.83 ± 0.16	38.57 ± 0.21	37.24 ± 0.53	37.87 ± 0.14	38.74 ± 0.34	39.24 ± 0.21	38.16 ± 0.14	37.66 ± 0.32	–	–
**BAT temperature (ºC)**		37.84 ± 0.26	38.21 ± 0.18	37.51 ± 0.50	37.27 ± 0.12	39.08 ± 0.19	**39.68 ± 0.17***	38.11 ± 0.20	38.07 ± 0.37	37.15 ± 0.29	**38.25 ± 0.21***
**Locomotor activity/lean mass (beam breaks/kg)**	**Light**	348.8 ± 15.1	396.0 ± 39.9	193.9 ± 43.4	221.5 ± 19.2	644.2 ± 49.6	611.9 ± 37.7	166.8 ± 10.1	167.5 ± 13.2	190.9 ± 16.4	184.6 ± 13.16
	**Dark**	838.7 ± 94.4	1105.5 ± 92.6	357.2 ± 62.0	638.2 ± 130	1932 ± 75.4	1928 ± 132.5	579.5 ± 76.5	432.1 ± 63.3	745.6 ± 132.2	525.8 ± 60.29
