# Supplementary material for: Vav2 catalysis-dependent pathways contribute to skeletal muscle growth and metabolic homeostasis
Source: Nat Commun. 2020 Nov 16;11:5808. doi: 10.1038/s41467-020-19489-z (PMC7669868; doi:10.1038/s41467-020-19489-z)
Supplement: Supplementary file 3 — Reporting Summary [file 41467_2020_19489_MOESM3_ESM.pdf]

nature research

Corresponding author(s): Xosé R. Bustelo

Last updated by author(s): Sep 14, 2020

Reporting Summary

Nature Research wishes to improve the reproducibility of the work that we publish. This form provides structure for consistency and transparency in reporting. For further information on Nature Research policies, see Authors & Referees and the Editorial Policy Checklist .

Statistics

For all statistical analyses, confirm that the following items are present in the figure legend, table legend, main text, or Methods section.

n/a	Confirmed	
☐	☒	The exact sample size (n) for each experimental group/condition, given as a discrete number and unit of measurement
☐	☒	A statement on whether measurements were taken from distinct samples or whether the same sample was measured repeatedly
☐	☒	The statistical test(s) used AND whether they are one- or two-sided<br>*Only common tests should be described solely by name; describe more complex techniques in the Methods section.*
☐	☒	A description of all covariates tested
☐	☒	A description of any assumptions or corrections, such as tests of normality and adjustment for multiple comparisons
☐	☒	A full description of the statistical parameters including central tendency (e.g. means) or other basic estimates (e.g. regression coefficient) AND variation (e.g. standard deviation) or associated estimates of uncertainty (e.g. confidence intervals)
☐	☒	For null hypothesis testing, the test statistic (e.g. F, t, r) with confidence intervals, effect sizes, degrees of freedom and P value noted<br>*Give P values as exact values whenever suitable.*
☒	☐	For Bayesian analysis, information on the choice of priors and Markov chain Monte Carlo settings
☒	☐	For hierarchical and complex designs, identification of the appropriate level for tests and full reporting of outcomes
☒	☐	Estimates of effect sizes (e.g. Cohen's d, Pearson's r), indicating how they were calculated

Our web collection on statistics for biologists contains articles on many of the points above.

Software and code

Policy information about availability of computer code

Data collection	No software was used for data collection.
Data analysis	GraphPad Prism software (version 6.0 and 8.3.1). LAS AF software (version 2.6.0.72266, Leica). StepOne software v2.1 (Applied Biosystems). ImageJ software (version 2.0.0-rc-69/1.52p; National Institutes of Health, Bethesda, MD).

For manuscripts utilizing custom algorithms or software that are central to the research but not yet described in published literature, software must be made available to editors/reviewers. We strongly encourage code deposition in a community repository (e.g. GitHub). See the Nature Research guidelines for submitting code & software for further information.

Data

Policy information about availability of data

All manuscripts must include a data availability statement. This statement should provide the following information, where applicable:

- Accession codes, unique identifiers, or web links for publicly available datasets
- A list of figures that have associated raw data
- A description of any restrictions on data availability

The datasets generated during the current study are available from the corresponding author upon reasonable request.

Field-specific reporting

Please select the one below that is the best fit for your research. If you are not sure, read the appropriate sections before making your selection.

☒ Life sciences ☐ Behavioural & social sciences ☐ Ecological, evolutionary & environmental sciences

For a reference copy of the document with all sections, see nature.com/documents/nr-reporting-summary-flat.pdf

Life sciences study design

All studies must disclose on these points even when the disclosure is negative.

Sample size	No statistical methods were used to determine sample size. In general, at least three independent replicates were performed in all experiments. For experiments subjected to higher variability, such as metabolic analyses, a larger number of animals was used. When possible, we have aimed for the replication of the animal experiments in at least two different cohorts. The sample size used for each experiment is indicated at the corresponding figure legend in the manuscript.
Data exclusions	Only significant outliers were excluded from the analysis. In figure 7c, one Vav2 L33A/L332A animal was excluded (0 min time-point). In figure S16c, one CD-fed WT was excluded in the 0 min time-point. In figure 7k, the 120 min time-point of a WT animal was excluded (lower than the 90 min time-point for the same mouse and significantly lower than the other animals). In figure 1a, one WT mice was excluded due to its low lean mass and abnormal metabolic parameters. In figure S11c, a 2-month-old Vav2 L332A/L332A animal was excluded due to its high adiposity content. In Fig. 8A, a WT animal had to be excluded from the final analysis and sacrificed due to health problems.
Replication	The number of independent replicates for each experiment is indicated at the corresponding figure legend in the manuscript. In general, at least three independent replicates were performed.
Randomization	In all cell and animal studies, groups were allocated randomly. Age and gender-matched animals were used in all the experiments.
Blinding	For all animal studies, the investigators were blind to group allocation. Blinding was not applicable to the rest of experiments.

Reporting for specific materials, systems and methods

We require information from authors about some types of materials, experimental systems and methods used in many studies. Here, indicate whether each material, system or method listed is relevant to your study. If you are not sure if a list item applies to your research, read the appropriate section before selecting a response.

Materials & experimental systems

n/a	Involved in the study	
☐	☒	Antibodies
☐	☒	Eukaryotic cell lines
☒	☐	Palaeontology
☐	☒	Animals and other organisms
☒	☐	Human research participants
☒	☐	Clinical data

Methods

n/a	Involved in the study	
☒	☐	ChIP-seq
☐	☒	Flow cytometry
☒	☐	MRI-based neuroimaging

Antibodies

Antibodies used	The antibodies used are described in the Methods section of the manuscript.<br>- Antibodies used in Western Bot: phospho-Akt (Thr308) (dilution 1:1000; Cat. #4056, Cell Signaling Technologies), Akt (dilution 1:1000; Cat. #2920, Cell Signaling Technologies), phospho-GSK3α/β (dilution 1:1000; Cat. #9327, Cell Signaling Technologies), GSK3α/β (dilution 1:1000; Cat. #5676, Cell Signaling Technologies), phospho-S6K (Thr389) (dilution 1:1000; Cat. #9215, Cell Signaling Technologies), phospho-ERK1/2 (Thr202/Tyr204) (dilution 1:1000; Cat. #4370, Cell Signaling Technologies), tubulin (dilution 1:2000; Cat. #CP06, Calbiochem), MHCII (dilution 1:2000; Cat. #M4276; Sigma), S6K (dilution 1:1000; Cat. #sc-230, Santa Cruz Biotechnologies), HA (dilution 1:1000; Cat. #3724, Cell Signaling Technologies), phospho-Tyr (for detecting phospho-IRS1; 1:1000 dilution, Cat. #sc-7020, Santa Cruz Biotechnologies), IRS1 (dilution 1:1000; Cat. #05-1085, Millipore), Rac1 (dilution 1:1000; Cat. #ARC03, Cytoskeleton), GFP (dilution 1:200; Cat. #902601, BioLegend), and Ucp1 (dilution 1:500; Cat. #sc-293418, Santa Cruz Biotechnologies). The polyclonal rabbit antibody to Vav2 (dilution 1:1000) was homemade using as epitope the acidic region of this protein.<br>- Antibodies used in immunofluorescence: antibody to the Myc epitope (Cat. #M5546, Sigma-Aldrich; 1:1000 dilution), and MHCII (1:700, Cat. #M4276; Sigma-Aldrich).
Validation	Commercially available antibodies (see above) have been validated by the manufacturer for the application (immunoblot, immunoprecipitation or immnunocytochemistry) and species. This information is available at each manufacturer's website and can be obtained through the catalog numbers indicated above. The homemade Vav2 antibody has been validated by us in overexpression, knockdown and knockout experiments.

Eukaryotic cell lines

Policy information about cell lines

Cell line source(s)	HEK293 (ATCC) and C2C12 (ATCC, obtained from Pura Muñoz-Canoves)

Authentication	ATCC cell lines are authenticated by the manufacturer. No additional authetication procedures were performed.
Mycoplasma contamination	All cell lines tested negative for mycoplasma contamination.
Commonly misidentified lines (See ICLAC register)	No commonly misidentified cell lines were used in this study.

Animals and other organisms

Policy information about studies involving animals; ARRIVE guidelines recommended for reporting animal research

Laboratory animals	Mus musculus. C57BL/6 and C57BL/10 backgrounds, both females and males (insulin and IGF infusion experiments) or males only (rest) were used with ages ranging between 2 and 12 months. The genotype (Vav2Onc/Onc, Vav2–/–, Vav2L332A/L332A or their correspondent WT) and age of the animals used in each experiment is detailed in the figure legends and the Methods section of the manuscript. Animals were kept in ventilated rooms in pathogen–free facilities under controlled temperature (23ºC), humidity (50%), and illumination (12–hour–light/12–hour–dark cycle) conditions.
Wild animals	No wild animals were used in this study.
Field-collected samples	No field-collected samples were used in this study.
Ethics oversight	Approved by the Bioethics Committees of the Universities of Salamanca, Santiago de Compostela and Geneva, as approppiate.

Note that full information on the approval of the study protocol must also be provided in the manuscript.

Flow Cytometry

Plots

Confirm that:

- [x] The axis labels state the marker and fluorochrome used (e.g. CD4-FITC).
- [x] The axis scales are clearly visible. Include numbers along axes only for bottom left plot of group (a 'group' is an analysis of identical markers).
- [x] All plots are contour plots with outliers or pseudocolor plots.
- [x] A numerical value for number of cells or percentage (with statistics) is provided.

Methodology

Sample preparation	The protocol is detailed in the Methods section of the manuscript.
Instrument	BD FACS Aria II (BD Biosciences)
Software	FlowJo 8.7.3
Cell population abundance	The abundance of the stem cell population in the Sca1-/CD45- population was around 5%.
Gating strategy	After a first gating using FSC-A/SSC-A, only the Sca1-/CD45- population was gated. The CD34+/Integrin alpha7+ population was considered the satellite cell population.

- [x] Tick this box to confirm that a figure exemplifying the gating strategy is provided in the Supplementary Information.
